# HnRNPA2B1 tunes antimycobacterial immune responses in macrophages through alternative splicing of *Irgm1*

**DOI:** 10.1128/iai.00732-25

**Published:** 2026-06-18

**Authors:** M. J. Chapman, J. B. Huskey, K. S. Armijo, S. Hahn, J. Spellman-Reliford, A. K. Coleman, C. J. Mabry, S. Carpenter, R. O. Watson, K. L. Patrick

**Affiliations:** 1 Department of Microbial Pathogenesis and Immunology, Texas A&M University, Naresh K. Vashist College of Medicine 12332, Bryan, Texas, USA; 2 Department of Pathology, Microbiology, and Immunology, Division of Molecular Pathogenesis, Vanderbilt University Medical Center 12328, Nashville, Tennessee, USA; 3 Department of Medicine, Division of Infectious Diseases, Vanderbilt University Medical Center 12328, Nashville, Tennessee, USA; 4 Department of Molecular, Cell, and Developmental Biology, University of California Santa Cruz 8787, Santa Cruz, California, USA; Rutgers New Jersey Medical School, Newark, New Jersey, USA

**Keywords:** RNA splicing, autophagy, antibacterial immunity, macrophages, innate immunity, host-pathogen interactions

## Abstract

The onset and progression of active tuberculosis disease result from upsetting the delicate balance between Mtb virulence and host defenses. Because it dynamically tunes the functional output of protein expression in cells, alternative splicing (AS), a process by which different mRNAs can be generated from a single gene, is positioned to play a critical role in maintaining an equilibrated Mtb-macrophage host-pathogen interface. To gain insight into how alternative splicing shapes antimycobacterial immune responses, we used RNA sequencing and splicing-aware computational pipelines to quantify alternative splicing in Mtb-infected bone marrow-derived murine macrophages. We found that ~5% of expressed macrophage genes exhibit one or more splicing changes at 8 h post-Mtb infection, highlighting alternative splicing as a key regulatory node in the macrophage response to Mtb. We next sought to identify RNA-binding proteins that play an outsized role in shaping the macrophage transcriptome during Mtb infection. We discovered that the splicing factor heterogeneous nuclear ribonucleoprotein A2B1 (hnRNPA2B1) promotes the early induction of inflammatory genes while dampening several type I interferon-stimulated genes in response to Mtb. HnRNPA2B1 also controls alternative splicing of many genes during Mtb infection, including Irgm1, a critical immunity-related GTPase. The balance of Irgm1-long vs. -short is differentially regulated in response to diverse inflammatory cues, and macrophages overexpressing Irgm1-short are defective in autophagosomal targeting, lysosomal homeostasis, and restriction of Mtb replication. These data highlight a key role for AS in shaping the macrophage transcriptome and pinpoint hnRNPA2B1 as a novel restriction factor in the cell-intrinsic response to Mtb.

## INTRODUCTION

Upon sensing pathogens like *Mycobacterium tuberculosis* (Mtb), macrophages rapidly reprogram their transcriptomes to engage antimicrobial defenses and alert neighboring cells to a newly detected threat. This response is initiated by pattern recognition receptors (PRRs), which detect specific pathogen-associated molecular patterns (PAMPs) in distinct subcellular compartments. PAMP recognition by PRRs triggers signaling cascades that activate transcription factors such as IRF3, AP-1, and NF-κB, which, in turn, drive rapid induction of immune response genes (1). The flexibility and specialization of PRR signaling allow macrophages to tailor responses to different pathogens. Mtb is sensed by multiple PRRs at different stages of infection, with TLR1/2/6 sensing of Mtb outer membrane glycoproteins and mycolic acids occurring at the plasma membrane and cGAS sensing of Mtb dsDNA occurring in the cytosol, following destabilization of the Mtb-containing phagosome (2–4). Together, these compartment-specific sensing pathways allow macrophages to integrate multiple microbial cues and calibrate their antimicrobial programs.

In addition to the activation of transcription, post-transcriptional regulatory steps, such as 3′ end formation and polyadenylation (5, 6), mRNA decay (7–10), and pre-mRNA splicing (11–18), are increasingly appreciated as critical determinants in shaping the innate immune milieu. Among these, splicing stands out because of its ubiquity and potential for diversifying the proteome. In the murine and human genomes, ~90% of protein-coding genes contain multiple exons (19), and ~95% of multi-exon genes are predicted to undergo alternative splicing (AS) (20, 21), a process whereby exons are selectively included or excluded in a regulated manner (22, 23). Through AS, a single gene can generate distinct isoforms from a common pre-mRNA, thereby expanding proteomic diversity independently of transcriptional activity (24–27).

Splicing decisions (where and when to splice) are controlled by RNA-binding proteins, particularly factors in the SR (serine-arginine rich) and hnRNP (heterogeneous nuclear ribonucleoprotein) families (22, 23). Generally, SR proteins promote splicing and exon inclusion, while hnRNPs inhibit splicing, causing exon skipping. SRs and hnRNPs have gained recent attention as orchestrators of immune cell reprogramming (11, 12, 16, 28–31). In macrophages, hnRNP M negatively regulates the abundance of inflammatory transcripts like *Il6* by slowing intron removal (12), and SRSF6 limits tonic interferon signaling by controlling alternative splicing of the mitochondrial pore-forming protein Bax (11). Splicing factors also have emerging roles in T cell immunity, where the SR protein Tra2b regulates *TCR* transcript splicing to decide T cell fate (28), and SRSF1 regulates splicing of *Cd6*, a protein involved in the coordination of antigen presentation (32). Together, these studies demonstrate that immune cells rely on splicing factors to execute specific responses to pathogens and/or inflammatory cues.

Earlier studies have established that Mtb infection elicits hundreds of AS events in human macrophages in functionally diverse genes, including *Il12rb1* (involved in dendritic cell migration), *Rab8b* (important for phagolysosomal maturation), and *Pgk1* and *Acsl1* (genes involved in glycolysis and lipid synthesis) (33, 34). AS events have also been identified as potential biomarkers for tuberculosis infection in human sera (35). Mechanistically, the inputs that influence AS during Mtb infection are likely multifactorial, with growing evidence that immune stimuli and Mtb secreted proteins can influence splice site selection (11, 36–39).

We recently became interested in a splicing factor called hnRNPA2B1. HnRNPA2B1 is an hnRNP A/B family member closely related to hnRNPA1 (40). HnRNPA2B1 has four annotated domains: an RNA-binding domain composed of two tandem RNA recognition motifs, an RGG box, a low complexity prion-like domain, and a PY-motif containing an M9 nuclear localization signal (PY-NLS) (41). HnRNPA2B1 plays known roles in neurologic development (42) and cancer (43–46) but remains understudied in the context of immunity. Outside of its role in splicing, hnRNPA2B1 can also act as a reader of mRNAs methylated by METTL3 (47–49), shuttle microRNAs in extracellular vesicles (50, 51), and protect transcripts from degradation (52, 53). Leveraging an hnRNPA2B1 conditional knockout mouse (cKO, myeloid lineage), we previously found that A2B1 is required to induce inflammatory mediators during endotoxin shock and *Salmonella* infection (29). Mice lacking A2B1 exhibited higher levels of CFUs in peripheral immune organs and had reduced survival compared to WT controls (29). Similar phenotypes were reported for A2B1 cKO mice infected with enterohemorrhagic *E. coli* and *Listeria monocytogenes* (54). Despite these compelling findings, the role of A2B1 in cell-intrinsic innate immunity remains unexplored.

Here, we integrate transcriptomic and molecular analyses to implicate A2B1 in host cell splicing changes during Mtb infection. We show that A2B1 plays a role in controlling AS of the immunity-related GTPase Irgm1. We report that only the long isoform of Irgm1 promotes restriction of Mtb in macrophages and that the balance of the two *Irgm1* isoforms is differentially regulated in response to diverse inflammatory cues. These findings underscore the importance of AS in shaping antimycobacterial immunity and motivate further studies of functionally distinct roles of AS isoforms at the host-pathogen interface.

## RESULTS

### Mtb induces alternative splicing of functionally diverse genes during macrophage infection

To date, AS has been implicated in the macrophage immune response to bacterial (*Salmonella enterica*, *Listeria monocytogenes*, and *Mycobacterium tuberculosis* [Mtb]) and viral (DENV, HIV, and Zika) pathogens (36, 55). Because macrophages serve as a replicative niche for Mtb and because AS transcripts have been identified as potential biomarkers for tuberculosis disease states in humans (35), we set out to refine our understanding of AS during Mtb infection of macrophages. Briefly, we infected bone marrow-derived macrophages (BMDMs) isolated from C57BL/6 mice with Mtb (Erdman, MOI = 5) and isolated total RNA at 8 h post-infection, a time point at which pattern recognition receptors and Mtb-responsive signaling cascades are fully engaged, but a few infected cells have initiated programmed cell death pathways. We performed bulk RNA-seq (see Materials and Methods) and profiled AS changes alongside uninfected controls, using the annotation-agnostic MAJIQ-VOILA framework (56). Significant local splice variations (LSVs) were defined by a dPSI >0.15 at a probability cutoff of 0.9 and a baseMean locus coverage >50. Given our interest in how splicing impacts immune protein expression, we applied a protein-coding gate to our pipeline, where any gene of interest needed to be able to produce at least one protein-coding isoform as determined by our ORF scanner and CPAT (coding probability > 0.5). To our knowledge, this represents one of the first reports of Mtb-induced AS in primary macrophages or in murine macrophages, providing an important foundation to explore the role of splicing in pre-clinical models of tuberculosis.

**Fig 1:**
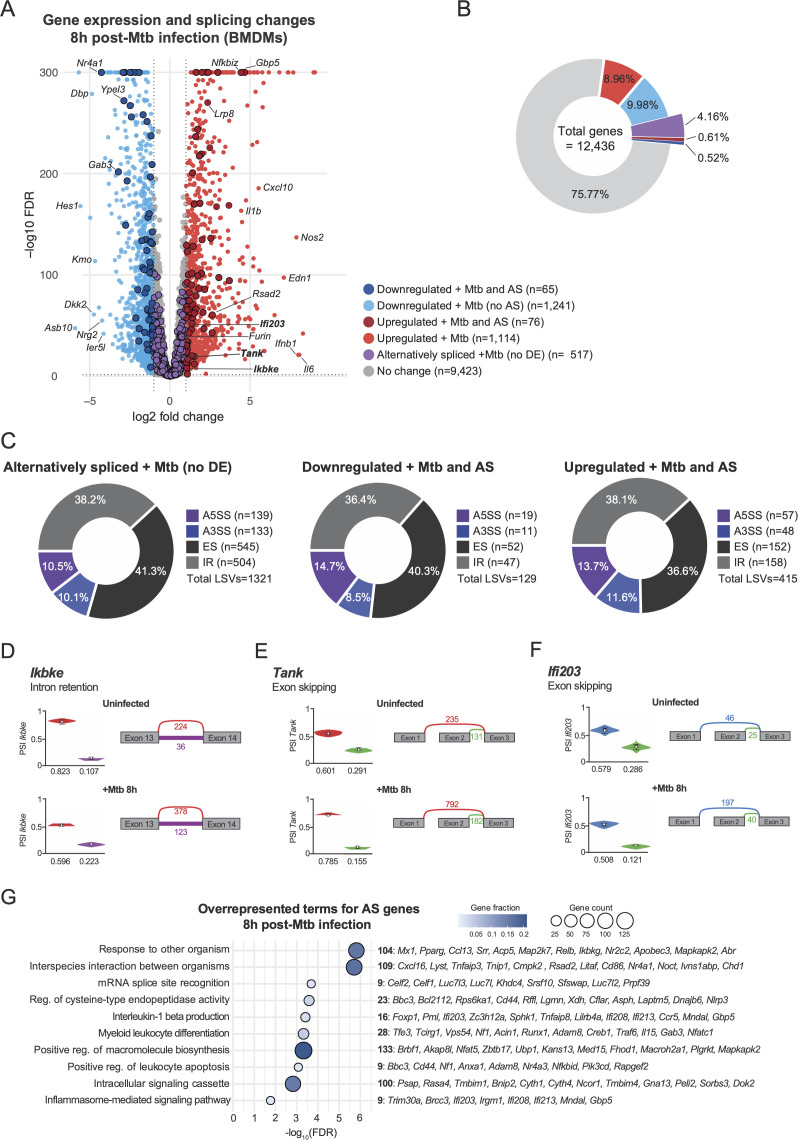
Mtb induces alternative splicing of functionally diverse genes during macrophage infection. (**A**) Volcano plot of differentially expressed genes (DEG) 8 hours post-Mtb infection in WT BMDMs, genes with at least one significant (dPSI > 0.15, confidence threshold > 0.90) AS event are darkened. (**B**) Pie charts of distribution of categories of DEG and AS. (**C**) Pie charts of distribution of categories of significant local splice variations for each DEG category. (**D**) MAJIQ PSI quantification and VOILA visualization of *Ikbke* junctions in uninfected (top) and Mtb-infected (bottom) BMDMs. (**E**) As in panel D, but for *Tank.* (**F**) As in panel D, but for *Ifi203.* (**G**) Over-representation analysis (ORA) of pathways enriched for AS genes between uninfected and Mtb-infected WT BMDMs. Statistical tests: RNA-seq differential expression (**A and C**) was performed using DESeq2 with Wald test statistics and Benjamini-Hochberg correction for multiple comparisons. Genes were considered differentially expressed at adjusted *P* < 0.05. Alternative splicing events (**A and C**) were identified using MAJIQ with a threshold of ΔPSI > 0.15 and probability > 0.90. Over-representation analysis (**G**) was performed using a hypergeometric test with false discovery rate correction. Data are derived from *n* = 3 biologically independent samples unless otherwise noted.

Of the 12,436 genes expressed in our BMDMs, MAJIQ-VOILA identified significant LSVs in ~5% of genes (658 protein-coding genes of 688 detected), spanning the four canonical AS event types: alternative 5′ splice site (A5SS), alternative 3′ splice site (A3SS), exon skipping (ES), and intron retention (IR) (Fig. 1A; Table S1). ES (40.2%) and IR (38.0%) were preferred LSVs, with fewer A5SS (11.5%) and A3SS (10.3%) events detected (Fig. S1A; Table S1). Many genes had multiple AS changes, with an average of 2.8 LSVs/gene (1,865 LSVs across 658 genes). Although many genes were transcriptionally up- or down-regulated during Mtb infection, most AS genes were not differentially expressed. Of the total 658 AS genes identified, 517 (4.16% of total expressed genes) underwent AS without differential expression, 65 (0.52%) underwent AS with decreased expression, and 76 (0.61%) underwent AS with increased expression (Fig. 1A and B; Table S1). The proportion of LSV type (ES, IR, A5SS, and A3SS) was similar between differential expression-status groups (Fig. 1C). Together, these data suggest that although some transcriptionally induced innate immune genes are subject to AS, macrophages also use AS to post-transcriptionally regulate constitutively expressed genes in response to Mtb infection.

To identify genes that undergo the most dramatic shifts in AS during Mtb infection, we manually annotated genes with dPSI >30% in each LSV category. Canonical immune genes meeting this threshold include the antiviral GTPase *Mx1*, the interferon-inducible guanylate binding protein *Gbp5,* and the IL-15 receptor subunit *Il15ra* (Fig. S1B). Notable immune genes with evidence of Mtb-regulated exon skipping included *Ikbke* (non-canonical kinase of IRF3 [57]), *Tank* (scaffold protein involved in TBK1-IRF3 signaling axis [58]), and *Ifi203* (non-canonical STING-dependent dsDNA sensor [59]) (Fig. 1D through F). To unbiasedly identify functional pathways enriched for genes that undergo AS during Mtb infection, we used ORA referencing the GO: Biological Process term list (Fig. 1G). Among the top 10 terms (FDR < 0.05, minimum pathway overlap of 2 genes, dPSI >15% in any LSV category), eight were immune-related (e.g., *Response to other organism*, *Myeloid leukocyte differentiation*, and *Regulation of AIM2 inflammasome assembly*). Several of these pathways align with prior studies of Mtb-infected THP1-1 cells, which also measured AS in genes involved in the innate immune response and response to stress (34). AS of the inflammasome sensor *Nlrp3* was notable, as its activity is known to be regulated by AS, but not yet in the context of infection (60, 61). Consistent with AS-mediated specialization of housekeeping genes during macrophage activation, we observed AS enrichment in genes involved in basic cellular and homeostatic functions—such as macromolecule biosynthesis, mRNA splice site recognition, and developmental regulation (Fig. 1G). Enrichment profiles of AS genes and upregulated DEGs showed some overlap (Fig. S1C and D), hinting at dual regulation of genes in some immune pathways (e.g., *Interspecies interaction between organisms* and *Intracellular signaling cassette*) by AS and transcription. Collectively, these analyses reveal that Mtb infection of macrophages triggers a widespread, coordinated program of alternative splicing—driven largely by ES and IR—that is largely uncoupled from changes to total transcript abundance.

### The splicing factor hnRNPA2B1 regulates the expression of inflammatory and type I interferon genes during Mtb infection

**Fig 2:**
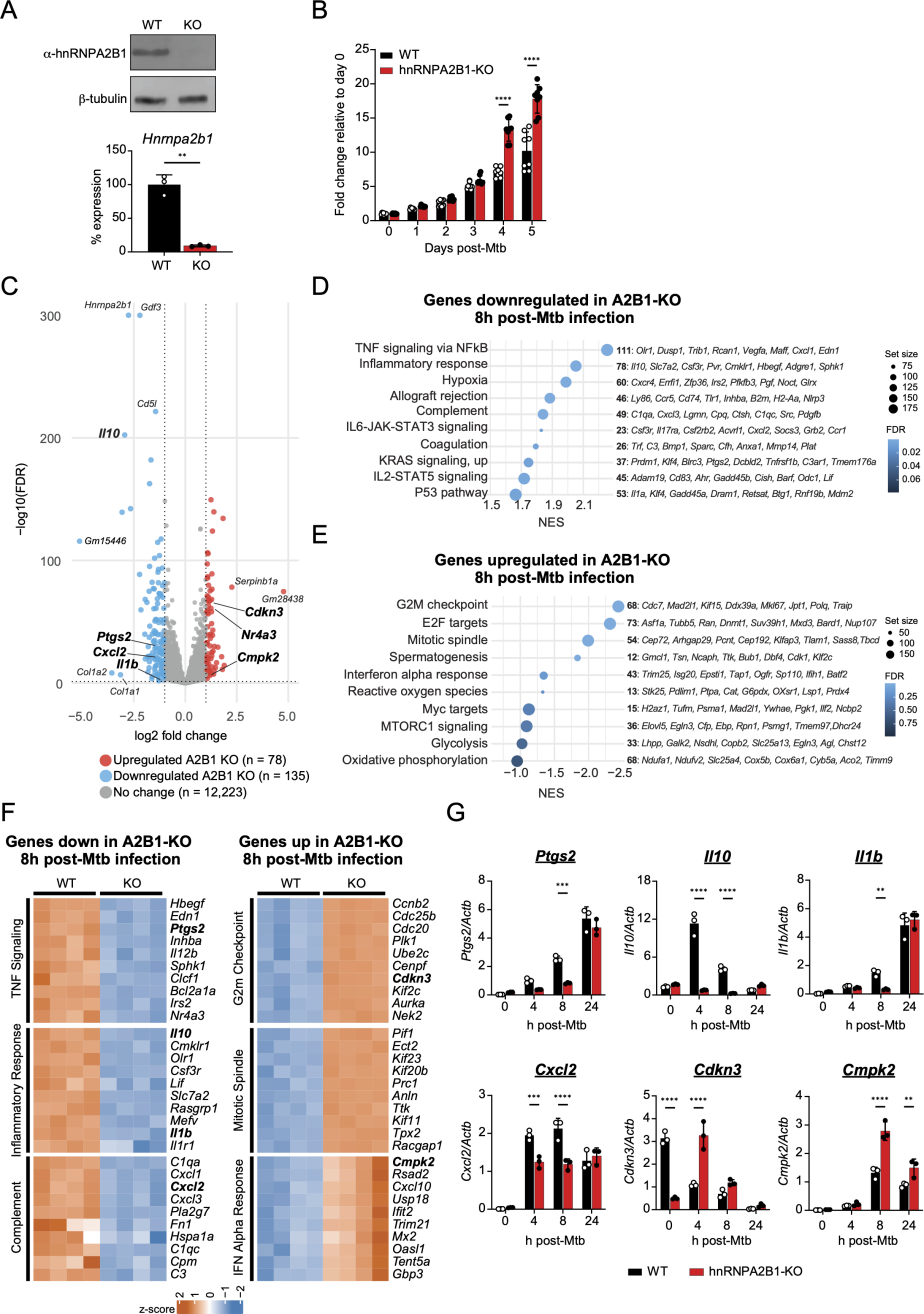
The splicing factor hnRNPA2B1 regulates the expression of inflammatory and type I interferon genes during Mtb infection. (**A**) Immunoblot and RT-qPCR of hnRNPA2B1 in WT and hnRNPA2B1-KO BMDMs. (**B**) Mtb replication in WT and hnRNPA2B1-KO BMDMs, measuring luminescence generated via Mtb-lux ABCDE, relative to day 0. n = 8. (**C**) Volcano plot of differentially expressed genes (DEGs) between WT and hnRNPA2B1-KO BMDMs 8 h post-Mtb infection. (**D**) Gene set enrichment analysis (GSEA) of pathways downregulated in hnRNPA2B1-KO BMDMs during Mtb infection compared to WT. (**E**) GSEA of pathways upregulated in hnRNPA2B1-KO BMDMs during Mtb infection compared to WT. (**F**) Heatmap of DEGs grouped by top HALLMARK terms in WT and hnRNPA2B1-KO BMDMs during Mtb infection. (**G**) RT-qPCR of selected DEGs in WT and hnRNPA2B1-KO BMDMs 0, 4, 8, and 24 h post-Mtb infection. n = 3. Statistical tests: Data are presented as mean ± SD from *n* = 3 biologically independent samples unless otherwise noted. For RT-qPCR experiments (**A and G**), statistical significance was determined using two-way ANOVA with multiple comparisons correction. Bacterial replication assays (**B**) were analyzed using two-way ANOVA with repeated measures. RNA-seq differential expression analysis (**C and F**) was performed using DESeq2 with Wald test statistics and Benjamini–Hochberg correction; genes were considered differentially expressed at adjusted *P* < 0.05. Gene set enrichment analysis (D–E) was performed using GSEA with significance determined by false discovery rate (FDR < 0.25). *P* < 0.05, *P < 0.01, **P < 0.001, ***P < 0.0001.

Our finding that distinct AS isoforms are generated in macrophages +/− Mtb hints that the process of splicing—and the factors that control it—could be subject to regulation during infection. RNA-binding proteins, particularly those in the SR and hnRNP families, play key roles in dictating splice site choice and toggling between AS isoforms (62). There is increasing appreciation that splicing factors themselves are functionalized during macrophage activation, such that they mediate the generation of specific AS isoforms that promote antimicrobial defenses (62). Based on our previous studies of the splicing factor hnRNPA2B1 in *Salmonella enterica*-infected mice, we hypothesized that A2B1 could play a role in the macrophage response to Mtb. To begin to test this, we harvested BMDMs from WT (LysM-cre/cre; hnRNPA2B1+/+) and KO (LysM-cre/cre; hnRNPA2B1 fl/fl) mice. Loss of A2B1 expression at the protein and RNA levels was confirmed via immunoblot and RT-qPCR, respectively (Fig. 2A). To establish A2B1 as a player in the macrophage response to Mtb, we first infected A2B1 KO and control BMDMs with Mtb-lux (Erdman strain) at an MOI = 1 and measured relative light units every 24 h for 5 days. We observed significantly higher Mtb-lux signal in A2B1 KOs compared to WT controls (Fig. 2B) without notable differences in cell death between the two genotypes (Fig. S2A), suggesting that A2B1 is a *bona fide* antibacterial restriction factor in macrophages. Motivated by this exciting result, we set out to better understand the molecular changes that could be driving this permissive phenotype. Briefly, WT and A2B1 KO BMDMs were infected with Mtb (MOI = 5, 8 h), and RNA was collected for RNA seq as shown in Fig. 1 (see Materials and Methods). Differential expression analysis revealed 78 genes upregulated and 135 downregulated in A2B1 KO cells relative to WT during Mtb infection (Fig. 2C). To gain insight into potential functional outcomes of these gene expression changes, we performed gene set enrichment analysis (GSEA) using the HALLMARK database to identify pathways enriched for downregulated genes (Fig. 2D) or upwnregulated genes (Fig. 2E) in A2B1 KO BMDMs compared to WT. We observed widespread dampening of inflammatory pathways in Mtb-infected A2B1 KOs BMDMs (e.g., *TNF signaling*, *Inflammatory response,* and *Complement*) (Fig. 2F; Fig. S2B-D), alongside upregulation of genes in pathways related to the cell cycle (*G2m Checkpoint* and *Mitotic Spindle*) and *Interferon alpha response* (Fig. 2D-F; Fig. S2E-G). RT-qPCR of representative genes in each HALLMARK category validated RNA-seq findings at 8 h post-Mtb infection (Fig. 2G; Fig. S2H). However, looking at a longer time course of Mtb infection, it appeared that transcriptional differences between genotypes were largely transient: differences between A2B1-KO and WT macrophages are evident at 4 h or 8 h, but not 24 h post-infection (Fig. 2G; Fig. S2H). Together, these findings suggest that hnRNPA2B1 plays a role in balancing the early induction of inflammatory and interferon-stimulated genes in response to Mtb.

### The splicing factor hnRNPA2B1 controls alternative splicing of genes during Mtb infection

**Fig 3:**
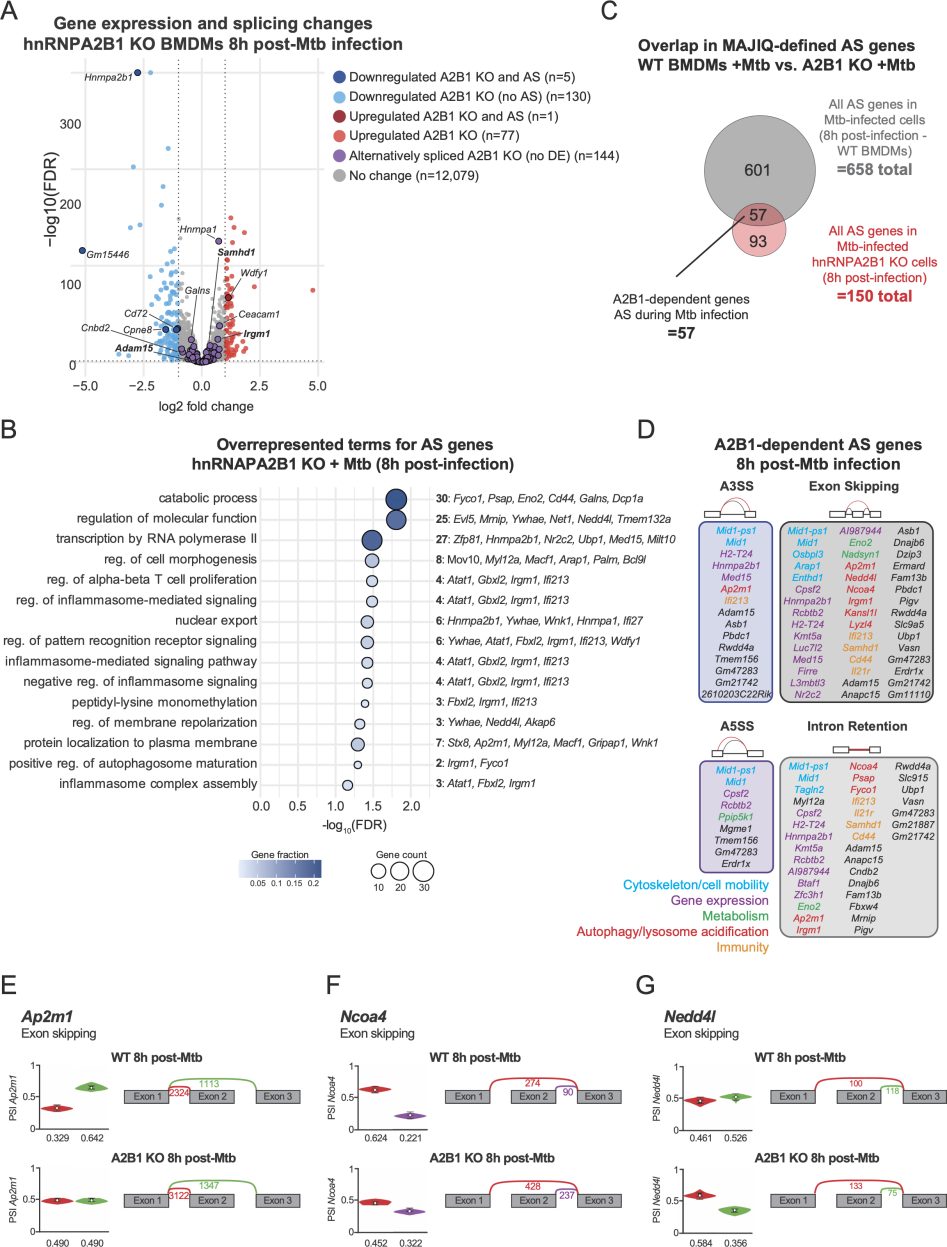
The splicing factor hnRNPA2B1 controls host alternative splicing decisions during Mtb infection. (**A**) Volcano plot of DEG between WT and hnRNPA2B1-KO BMDMs 8 h post-Mtb infection. Genes with at least one significant (dPSI > 0.15, confidence threshold > 0.90) AS event are darkened. (**B**) Over-representation analysis of pathways enriched for AS genes between WT and hnRNPA2B1-KO BMDMs 8 h post-Mtb infection. (**C**) Overlap of significant AS genes in the WT vs 8 h Mtb and 8 h Mtb WT vs. hnRNPA2B1-KO sets. (**D**) Lists of significant AS genes per LSV category from the overlap region in panel C. (**E**) MAJIQ PSI quantification and VOILA visualization of *Ap2m1* in WT (top) and A2B1 KO (bottom) Mtb-infected macrophages. (**F**) As in panel E but for *Ncoa4.* (**G**) As in panel E but for *Nedd4l.* Statistical tests: RNA-seq differential expression (**A**) was performed using DESeq2 with Wald test statistics and Benjamini–Hochberg correction; genes were considered differentially expressed at adjusted *P* < 0.05. Alternative splicing events (**A**, **C–G**) were identified using MAJIQ with a threshold of ΔPSI > 0.15 and probability > 0.90. Over-representation analysis (**B**) was performed using a hypergeometric test with false discovery rate correction. Data are derived from *n* = 3 biologically independent samples unless otherwise noted.

Next, we asked how hnRNPA2B1 impacts AS during Mtb infection (8 h). We identified 150 genes with significant AS changes (150 protein-coding genes, of 151 AS detected), of which 144 showed no corresponding change in gene expression (Fig. 3A; Table S3). ORA of these 150 A2B1-dependent AS genes uncovered that seven of the top 15 enriched pathways are related to the macrophage immune response (*Regulation of the inflammasome, PRR signaling,* and *Autophagosome maturation*) (Fig. 3B).

Comparison of A2B1-dependent AS events with those induced by Mtb in WT cells revealed 57 overlapping genes, representing Mtb infection-driven, hnRNPA2B1-dependent AS events. Most shared events were categorized as exon inclusion, consistent with the known role of A2B1 in promoting exon skipping (Fig. 3C). These hnRNPA2B1-dependent AS events occurred in genes involved in a variety of pathways, several with known links to antimycobacterial immunity (e.g., metabolism, autophagy, and lysosome acidification) (Fig. 3D). Notably, we detected A2B1-dependent exon skipping in *Ap2m1* (Fig. 3E), *Ncoa4* (Fig. 3F), and *Nedd4l* (Fig. 3G). *Ap2m1* encodes a component of the AP-2 clathrin adaptor complex that can be hijacked by *Listeria monocytogenes* to promote infection (63). *Ncoa4* is an E3 ubiquitin ligase involved in autophagy of ferritin-iron complexes (64). *Nedd4l* is an E3 ubiquitin ligase involved in autophagy, regulation of inflammation (65), and control of *Mycobacterium bovis* BCG replication (66). Collectively, these findings identify hnRNPA2B1 as a regulator of splicing decisions during Mtb infection, notably influencing isoform selection of genes acting in pathways with known links to mycobacterial immunity.

### *Irgm1* has two isoforms that are differentially regulated upon immune activation of macrophages

**Fig 4:**
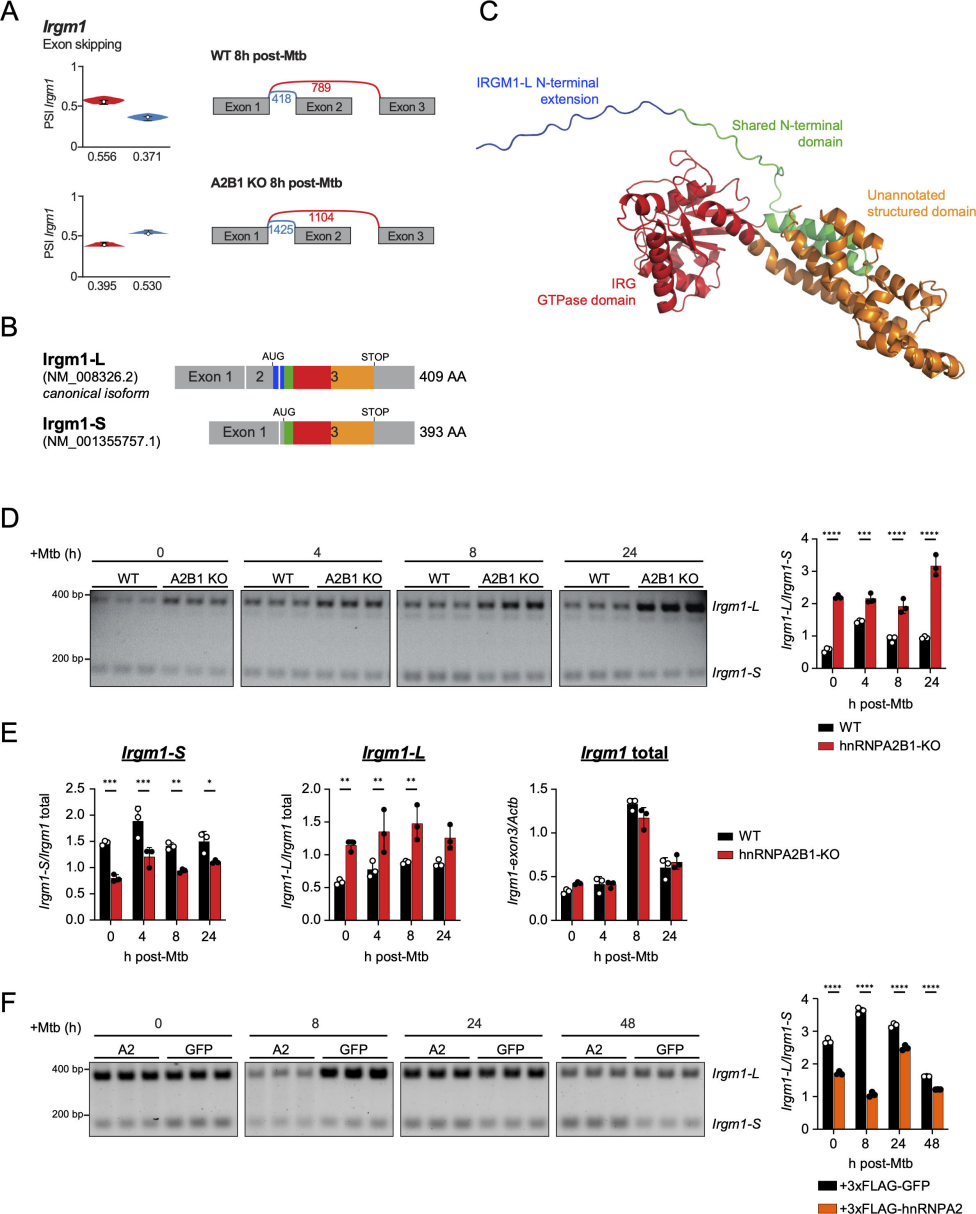
hnRNPA2B1 controls alternative splicing of Irgm1. (**A**) MAJIQ PSI quantification and VOILA visualization of *Irgm1* in WT (top) and hnRNPA2B1 KO (bottom) Mtb-infected BMDMs. (**B**) Diagram of Irgm1 isoform exon usage. (**C**) Predicted AlphaFold structure of Irgm1 with annotated regions highlighted. (**D**) Semi-quantitative RT-PCR of *Irgm1* exon 2 inclusion in WT and hnRNPA2B1-KO BMDMs 0, 4, 8, and 24 h post-Mtb infection. Quantification represented as band intensity of *Irgm1-long* (top) over band intensity of *Irgm1-short* (bottom), shown on the right. *n* = 3. (**E**) RT-qPCR of *Irgm1* isoforms and total transcript abundance in WT and hnRNPA2B1-KO BMDMs 0, 4, 8, and 24 h post-Mtb infection. n = 3. (**F**) Semi-quantitative RT-PCR of *Irgm1* exon 2 inclusion in GFP and hnRNPA2B1 overexpression iBMDMs 0, 8, 24, and 48 h post-Mtb infection. Quantification represented as band intensity of *Irgm1-long* (top) over band intensity of *Irgm1-short* (bottom), shown on the right. *n* = 3. Statistical tests: Data are presented as mean ± SD from *n* = 3 biologically independent samples unless otherwise noted. For semi-quantitative RT-PCR band intensity quantification (**D and F**) and RT-qPCR analyses (**E**), statistical significance was determined using two-way ANOVA with multiple comparisons correction. Alternative splicing events (**A**) were identified using MAJIQ with a threshold of ΔPSI > 0.15 and probability > 0.90. Structural prediction (**C**) was performed using AlphaFold. *P* < 0.05, *P < 0.01, **P < 0.001, ***P < 0.0001.

Among hnRNPA2B1-regulated AS genes, *Irgm1* emerged as a compelling hit due to its known roles in autophagy and antimycobacterial immunity (Fig. 4A). *Irgm1* (Immunity-Related GTPase family M protein 1) is a murine GTPase essential for host defense, with established roles in mitophagy, autophagy, and resistance to Mtb (67–69)*. Irgm1* is functionally orthologous to human *IRGM*, which shares the IRG-like GTPase domain (PS51716) (Fig. 4B and C; Fig. S3A). *Irgm1^-/-^* mice have been repeatedly shown to be incredibly sensitive to Mtb infection (68, 70, 71). While *in vivo* phenotypes have recently been attributed to misregulation of the type I IFN response (71), many foundational studies implicate Irgm1 in cell-intrinsic control of mycobacteria via autophagy (70–73). Relevant to our studies of AS in BMDMs, the murine *Irgm1* locus produces two protein-coding isoforms via exon 2 inclusion: Irgm1-long, which contains a 16-aa N-terminal extension, and Irgm1-short, which lacks this region. Prior work suggests that both isoforms can localize to the Golgi, but only Irgm1-long localizes to lysosomes (73). Beyond published studies that explore their subcellular localization (73), the two Irgm1 isoforms in mice remain functionally uncharacterized.

To validate our RNA-seq findings and examine the dynamics of Irgm1 AS, we used semi-quantitative RT-PCR to quantify the long isoform (contains exon 2; generates a 394 bp PCR product) and the short isoform (exon 2 is skipped; generates a 175 bp PCR product) in RNA isolated from WT and A2B1-KO BMDMs at 4, 8, and 24 h post-Mtb infection (Fig. S3B). Consistent with our RNA-seq data, A2B1 KO macrophages accumulated the long isoform in all time points relative to controls (Fig. 4D). RT-qPCR confirmed these results (Fig. 4E; Fig. S3C). Notably, total *Irgm1* transcript abundance remained unchanged between genotypes, indicating that A2B1 controls splicing rather than transcriptional regulation of this gene (Fig. 4E). Total protein levels of IRGM were also unchanged in A2B1 KO BMDMs compared to controls (Fig. S3D), although it is worth noting that commercially available antibodies that recognize murine IRGM cannot distinguish between Irgm1, 2, or 3 (or any of the isoforms encoded by these genes). We then asked if overexpression of A2B1 was sufficient to promote Irgm1-short expression. To this end, we transduced iBMDMs to stably express 3xFLAG-hnRNPA2 (the dominant form of A2B1 expressed in macrophages) or a 3xFLAG-GFP control (Fig. S3E) and again measured Irgm1-long vs. short by semi-quantitative RT-PCR. Baseline expression of Irgm1-long expression was elevated in both A2 and GFP-expressing cells, which we believe to be a consequence of slight macrophage activation following lentiviral transduction and selection. Regardless, following Mtb infection, we see a clear shift in *Irgm1* isoform usage in the A2-expressing cells, reinforcing a role for hnRNPA2B1 in promoting Irgm1 exon 2 exclusion (Fig. 4F). Collectively, these findings establish hnRNPA2B1 as a key regulator of *Irgm1* splicing in murine macrophages.

Previous reports have shown that Irgm1-long and -short differ in their subcellular localization (73). To further explore this, we fed the sequences of Irgm1-long and -short into DeepLoc2.1, a software that predicts the subcellular localization of proteins (74). Although the localization predictions did not meet the program’s significance threshold, DeepLoc2.1 predicts that Irgm1-short is most likely found in the cytosol and plasma membrane, whereas Irgm1-long is most likely targeted to lysosomes (Fig. S3F). Consistent with this output, we identified a canonical dileucine lysosomal targeting motif ([D/E]XXXLL) in the 16 amino acid N-terminal extension encoded in Irgm1-long (Fig. S3G) (75). Palmitoylation of Irgm1 has been shown to control its ability to associate with membranes, but this modification was found in a C-terminal amphipathic helix that is shared between Irgm1-long and short. Thus, we would not expect this site to mediate differential targeting of the isoforms (76).

To test the DeepLoc2.1 predictions, we transfected N-terminal FLAG-tagged Irgm1-long*,* Irgm1-short*,* or a GFP overexpression control into HEK293T cells (Fig. S3H) and monitored subcellular localization of each construct via immunofluorescence microscopy. In our hands, enrichment of 3*×*FLAG-Irgm1-long in HEK293Ts was observed in what look like vesicular structures throughout the cytoplasm (Fig. S3I). 3*×*FLAG-Irgm1-short, on the other hand, was enriched at the plasma membrane and in punctate/tubular structures through the cytosol. Attempts to colocalize either isoform with Tomm20+ (mitochondrial) or LAMP1+ (lysosomal) compartments yielded inconclusive results (data not shown), likely due to the high level of Irgm1 overexpression. Thus, although Irgm1-long and -short appear to be enriched in distinct cellular compartments, the nature of these compartments remains undefined. Overall, our results are consistent with published reports of IRGM proteins broadly labeling a variety of cellular compartments (e.g., Golgi, ER, phagosomes, endosomes, and mitochondria) as “self,” for protection against GKS-protein mediated damage (73, 77–80).

### Overexpression of *Irgm1-short* inhibits Mtb restriction in macrophages

**Fig 5:**
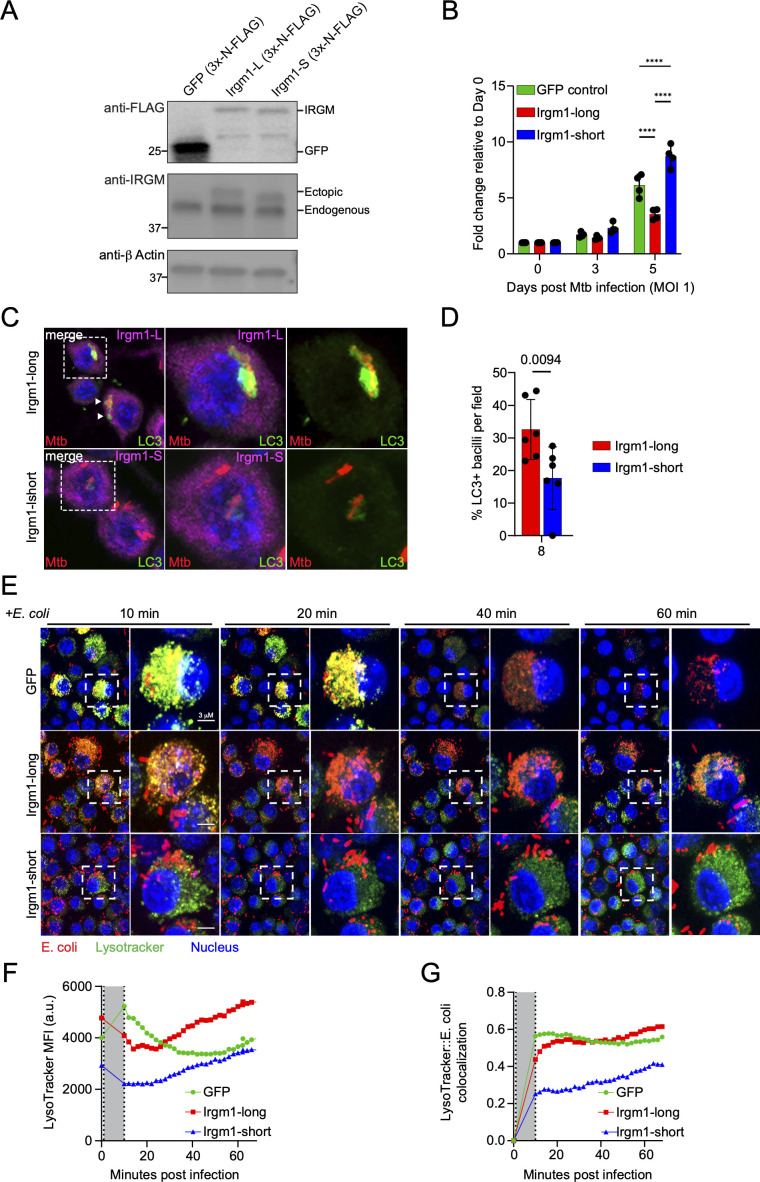
Irgm1 isoforms have different capacity for restricting Mtb replication in iBMDMs. (**A**) Immunoblot of IRGM in GFP, FLAG-Irgm1-long, and FLAG-Irgm1-short stably expressed in WT iBMDMs. (**B**) Fold replication of Mtb in GFP, Irgm1-long, or Irgm1-short stable expression in iBMDMs 0, 3, and 5 days post-infection, measuring luminescence generated via Mtb-lux ABCDE, relative to day 0. *n* = 4. (**C**) Immunofluorescence microscopy of Irgm1-isoforms (anti-FLAG, pink), LC3 (green), and Mtb (mCherry, red) 8 h post-Mtb infection in Irgm1-long and Irgm1-short-expressing iBMDMs. (**D**) Quantification of results shown in panel C. (**E**) Live-cell fluorescence microscopy of *E. coli* (mCherry, red), LysoTracker (green), and nucleus (blue) 0–68 min post-*E. coli* infection in GFP, Irgm1-long, and Irgm1-short iBMDMs. (**F**) Quantification of results shown in panel E. Total LysoTracker per field at baseline and through the duration of infection. Gray bar represents the infection step (10 minutes). (**G**) Quantification of results shown in panel E; Pearson colocalization coefficient of LysoTracker with mCherry at baseline and through the duration of infection. Gray bar represents the infection step (10 min). Statistical tests: Data are presented as mean ± SEM from *n* = 4 biologically independent samples unless otherwise noted. For bacterial replication assays (**B**), statistical significance was determined using two-way ANOVA with repeated measures and multiple comparisons correction. Quantification of immunofluorescence and live-cell imaging experiments (**D, F, and G**) was performed using one-way or two-way ANOVA, as appropriate, with multiple comparisons correction. Pearson colocalization coefficients (**G**) were calculated to assess spatial overlap between signals. Immunoblot (**A**) and representative microscopy images (**C and E**) are shown from independent experiments. *P* < 0.05, *P < 0.01, **P < 0.001, ***P < 0.0001.

To understand the functional consequences of *Irgm1* AS in the context of antimycobacterial immunity, we generated WT iBMDM cell lines that stably overexpress each isoform (Fig. 5A) and infected them with Mtb-lux, monitoring infectious burden via luciferase assay over 5 days (as in a previous study [81]). Relative to the GFP control, cells overexpressing Irgm1-long had lower bacillary burdens, whereas cells overexpressing Irgm1-short had higher burdens (Fig. 5B). To rule out the possibility that differences in Mtb-lux signal could be due to altered sensitivity of the two isoform-expressing cell lines to undergo cell death, we monitored propidium iodide incorporation over a 24 h time course of Mtb infection and found that cell death kinetics were similar in cells expressing GFP, Irgm1-long, and Irgm1-short (Fig. S4A). Likewise, monolayers remained intact over the entire 5-day time course (Fig. S4B), arguing that Irgm1-long restricts Mtb replication via a cell death-independent mechanism.

Given that the two isoforms differ in their ability to control Mtb survival/replication (Fig. 5B), and predictions that they may differentially localize with lysosomes (Fig. S3F and G), we reasoned that Irgm1-short isoform-expressing cell lines may be defective in autophagy, the process by which cells wall—off unwanted or damaged cargo, forming autophagosomes, which fuse with lysosomes to degrade cargo. Previous studies have repeatedly linked IRGM to autophagy at a number of steps, including activation of ULK1 and Beclin1, recruitment of autophagy proteins to growing autophagosomes, promoting fusion between the autophagosome and lysosomes, and stimulating lysosomal biogenesis (67).

To begin to test the involvement of Irgm1-long and -short in antimycobacterial autophagy, we used immunofluorescence microscopy to quantify abundance and localization of autophagy factors at 8 h post-infection of Irgm1 isoform-expressing iBMDMs with mCherry Mtb. Consistent with Mtb-lux results in Fig. 5B, we measured increased bacilli area in Irgm1-short iBMDMs at 24 h post-infection by quantifying mean fluorescence intensity measured across segmented bacillus area (Fig. S4C). We have previously shown that LC3 is recruited to a population of Mtb-containing phagosomes at 6–8 h post-infection and is degraded during lysosomal turnover of autophagosomal cargo (82). Early studies of IRGM1 from the Deretic lab demonstrated fewer LC3+ mycobacteria in Irgm1 knockdown macrophages (83). Consistent with this finding, we measured higher LC3 MFI per cell and a higher percentage of LC3+ Mtb bacilli in Irgm1-long expressing cells relative to Irgm1-short at 8 h post-infection (Fig. 5C and D; Fig. S4D). Because LC3+ membranes are turned over when autophagosomes fuse with lysosomes, fewer LC3+ bacilli in Irgm1-short iBMDMs could represent either impaired autophagosomal targeting or enhanced lysosomal degradation. To begin to distinguish these models, we quantified Mtb colocalization with the lysosomal marker, LAMP1. Although the total LAMP1 signal was the same in Irgm1-long vs. -short expressing iBMDMs (Fig. S4E), Irgm1-short cells showed significantly less LAMP1-Mtb colocalization 8 h post-infection (Fig. S4F), supporting a model whereby the expression of Irgm1-short impairs LC3+ tagging of Mtb, thereby reducing lysosomal targeting of autophagosomes.

Given that Irgm1-long is reported to colocalize with lysosomes (73) and our data hint at differences in LAMP1+ bacilli in Irgm1-long and -short expressing cells, we also wanted to investigate the role of Irgm1 isoforms in macrophage lysosomal biology. Because our BSL3 lacks live-cell imaging capabilities, we turned to another intracellular bacterial model. Briefly, we infected Irgm1-long, Irgm1-short, and GFP-expressing iBMDMs with a lab strain of mCherry-expressing *E. coli* (DH5a) and monitored the acidification of *E. coli*-containing compartments via live-cell imaging of LysoTracker. Because non-pathogenic *E. coli* has not evolved any strategies to avoid delivery to lysosomes, macrophages rapidly target bacilli to lysosomes for destruction. We found that cells expressing Irgm1-long had a higher LysoTracker MFI at baseline (Fig. S4G and H) and over the duration of the infection (Fig. 5E and F), as well as higher colocalization of LysoTracker with mCherry *E. coli* (Fig. 5E and G), compared to cells expressing Irgm1-short (Video S1). These findings suggest that Irgm1-short is sufficient to impair lysosomal function, via failure to maintain low pH compartments and by inhibiting fusion of lysosomes with bacteria-containing endosomes.

### Pathogen-derived and inflammatory stimuli differentially regulate *Irgm1* AS

**Fig 6:**
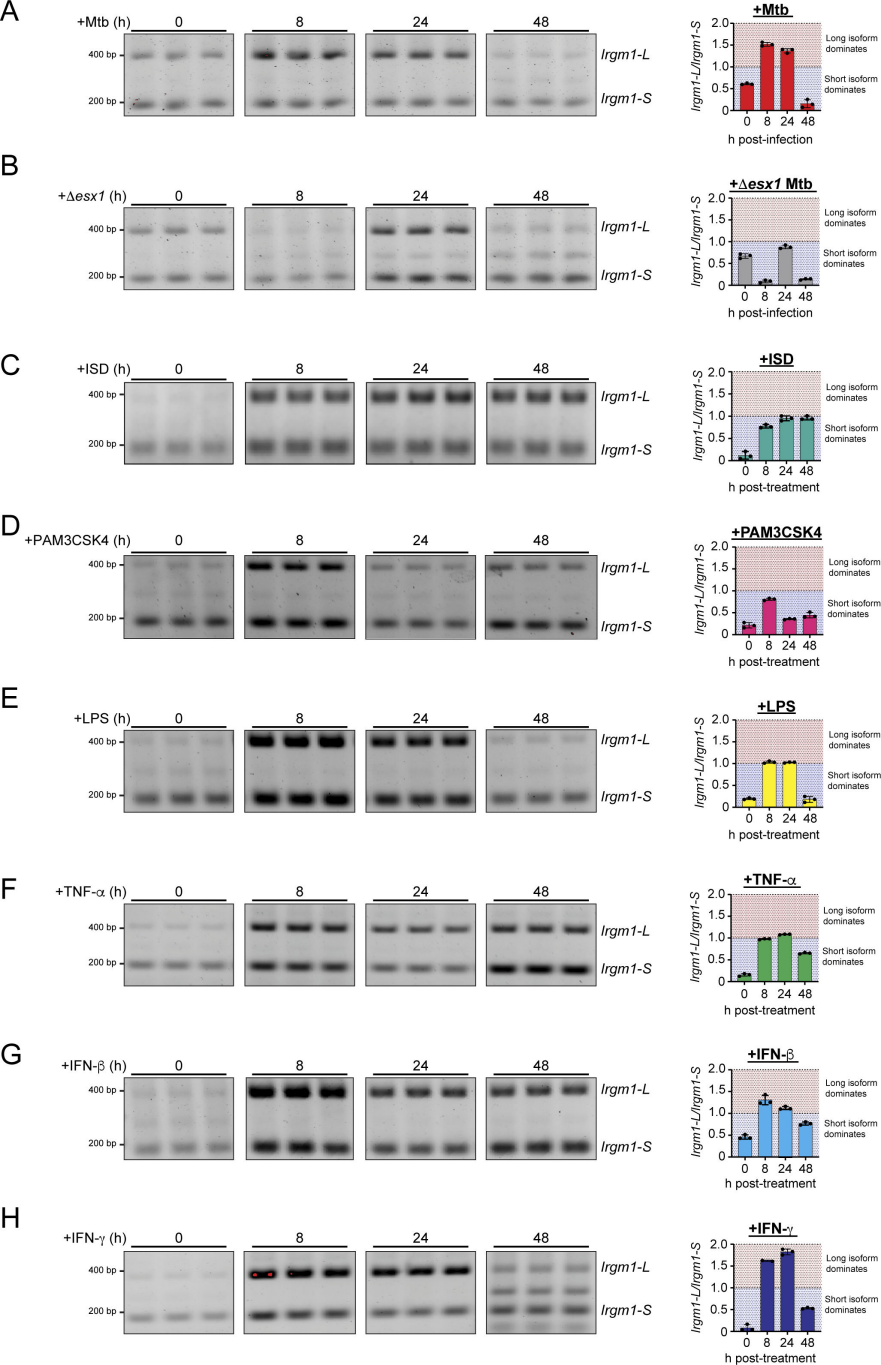
Irgm1 splicing in BMDMs is differentially responsive to distinct innate immune cues. (**A**) Semi-quantitative RT-PCR of *Irgm1* exon 2 inclusion in WT and hnRNPA2B1-KO BMDMs 0, 8, 24, and 48 h post-Mtb infection (MOI = 5). Quantification on the right is represented as the band intensity of *Irgm1-long* (top) over the band intensity of *Irgm1-short* (bottom). *n* = 3. (**B**) As in panel A, but post-infection with ΔESX1-Mtb (MOI = 5). (**C**) As in A, but post-transfection with ISD (1 μg/mL). (**D**) As in panel A, but post-stimulation with PAM3CSK4 (1 μg/mL). (**E**) As in panel A, but post-stimulation with LPS (10 ng/mL). (**F**) As in panel A, but post-stimulation with TNF-α (20 ng/mL). (**G**) As in panel A, but post-stimulation with IFN-β (50 IU/mL). (**H**) As in A, but post-stimulation with IFN-γ (50 IU/mL). Statistical tests: Data are presented as mean ± SD from *n* = 3 biologically independent samples unless otherwise noted. For semi-quantitative RT-PCR band intensity quantification (A–K), statistical significance was determined using two-way ANOVA with multiple comparisons correction. Comparisons of Irgm1-long to Irgm1-short ratios across genotypes, treatments, or time points were analyzed as indicated in each panel. *P* < 0.05, *P < 0.01, **P < 0.001, ***P < 0.0001.

Upon phagocytosis, Mtb engages the host macrophage through PRRs (TLR1/2/6, cGAS-STING) and through the release of virulence factors (via various secretion systems). After PRR engagement, infected macrophages release and respond to various cytokines, notably TNF-α, IL1-β, IFN-β, and IFN-γ. Given the functional consequences of the *Irgm1* alternative splicing, we set out to characterize how Mtb-relevant immune stimuli influence *Irgm1* splicing decisions. Using the same semi-quantitative RT-PCR approach employed in Fig. 4D, we first asked whether upregulation of Irgm1-long was dependent on Mtb’s ESX-1 secretion system, which controls cytosolic access and delivery of key virulence factors (84, 85). Compared to WT Mtb-infected BMDMs, which express high amounts of Irgm1-long relative to -short at 8 and 24 h post-infection (Fig. 6A), ΔESX-1 Mtb-infected macrophages make more of the short isoform (Fig. 6B), suggesting that Mtb secreted factors and/or cytosolic access of Mtb promote the generation of Irgm1-long during infection. Because treatment of Mtb-infected cells with the STING inhibitor H-151 had no effect on Irgm1 AS until very late time points (48 h post-infection), we do not think that early upregulation of Irgm1-long relies on cGAS-STING signaling (Fig. S5A, D, and E). Of note, higher baseline expression of Irgm1-long in uninfected samples (Fig. 6A and B) likely results from our use of media containing horse serum for our mock infection condition, which we suspect slightly activates macrophages.

We next assessed how individual activation of Mtb-relevant PRRs influences *Irgm1* splicing. Briefly, we transfected BMDMs with interferon stimulatory DNA (ISD) (cGAS agonist [1 µg/mL]) or stimulated BMDMs with PAM3CSK4 (TLR1/2/6 agonist [1 µg/mL]). Consistent with inhibiting STING during Mtb infection, we found that ISD promoted roughly equal amounts of *Irgm1-long* and *Irgm1-short* (Fig. 6C). We found that stimulation with PAM3CSK4 also promotes similar amounts of each isoform (Fig. 6D). Although not relevant during Mtb infection, we also asked how engagement of TLR4 via LPS altered *Irgm1* isoform abundance. Consistent with TLR4 and TLR2 signaling sharing many components, LPS (10 ng/mL) also promoted similar levels of Irgm1-long and -short (Fig. 6E).

Finally, we asked how different secreted cytokines, sensed in an autocrine or paracrine fashion by macrophages, impact *Irgm1* AS. TNF-α (20 ng/mL), an abundant pro-inflammatory cytokine that is rapidly induced upon Mtb infection of macrophages (86), promoted similar amounts of Irgm1-long and -short (Fig. 6F). Interestingly, although the antibacterial type II interferon IFN-γ (50 IU/mL) and the pro-bacterial type I IFN IFN-β (50 IU/mL) both transcriptionally upregulate *Irgm1* (72, 87), IFN-γ treatment was much better at promoting Irgm1-long relative to short (Fig. 6G and H). In fact, blocking IFNAR with a monoclonal antibody during Mtb infection was sufficient to push Irgm1 splicing toward the long isoform, suggesting that IFNAR signaling negatively regulates the generation of Irgm1-long (Fig. S5B, D, and E). Given that type I IFN has been repeatedly shown to promote Mtb pathogenesis in macrophages and *in vivo* (88–90), it is tempting to speculate that failure to generate Irgm1-long in a type I IFN-dominant cytokine milieu contributes to these phenotypes. Together, these data show that *Irgm1* AS can be differentially regulated in response to distinct inflammatory cues and suggest that IFN-γ, a potent antibacterial cytokine, is unique in its ability to promote the Mtb-restrictive isoform of Irgm1. Because no single agonist treatment recapitulated the Mtb-induced dynamics of Irgm1 isoform expression, our data also argue that the lack of colocalization between Eregulation of Irgm1 AS during Mtb infection involves multiple inputs.

## DISCUSSION

Tight control of antimicrobial proteins is required to preserve cellular homeostasis while maintaining the capacity to mount effective host defenses. The family of dynamin-like immunity-related GTPases (IRGs), of which Irgm1 is a member, is divided into two groups: GKS “effector proteins” and GMS “regulatory proteins.” The presence of GMS proteins like Irgm1 on intracellular membranes is thought to mark them as “self,” preventing the activation of GKS proteins and subsequent GKS-mediated damage (91, 92). To date, studies of Irgm1 in host defense and autoimmunity have largely focused on its canonical long isoform. Here, we demonstrate that AS plays an important role in toggling *Irgm1* between a long isoform and a short isoform that lacks an N-terminal domain. By implicating the splicing factor hnRNPA2B1 in balancing the abundance of Irgm1-long vs. short and showing that Irgm1-long abundance is enhanced by Mtb infection and IFN-γ treatment, this work advances our understanding of how macrophages use AS to regulate antimycobacterial innate immunity.

**Fig 7:**
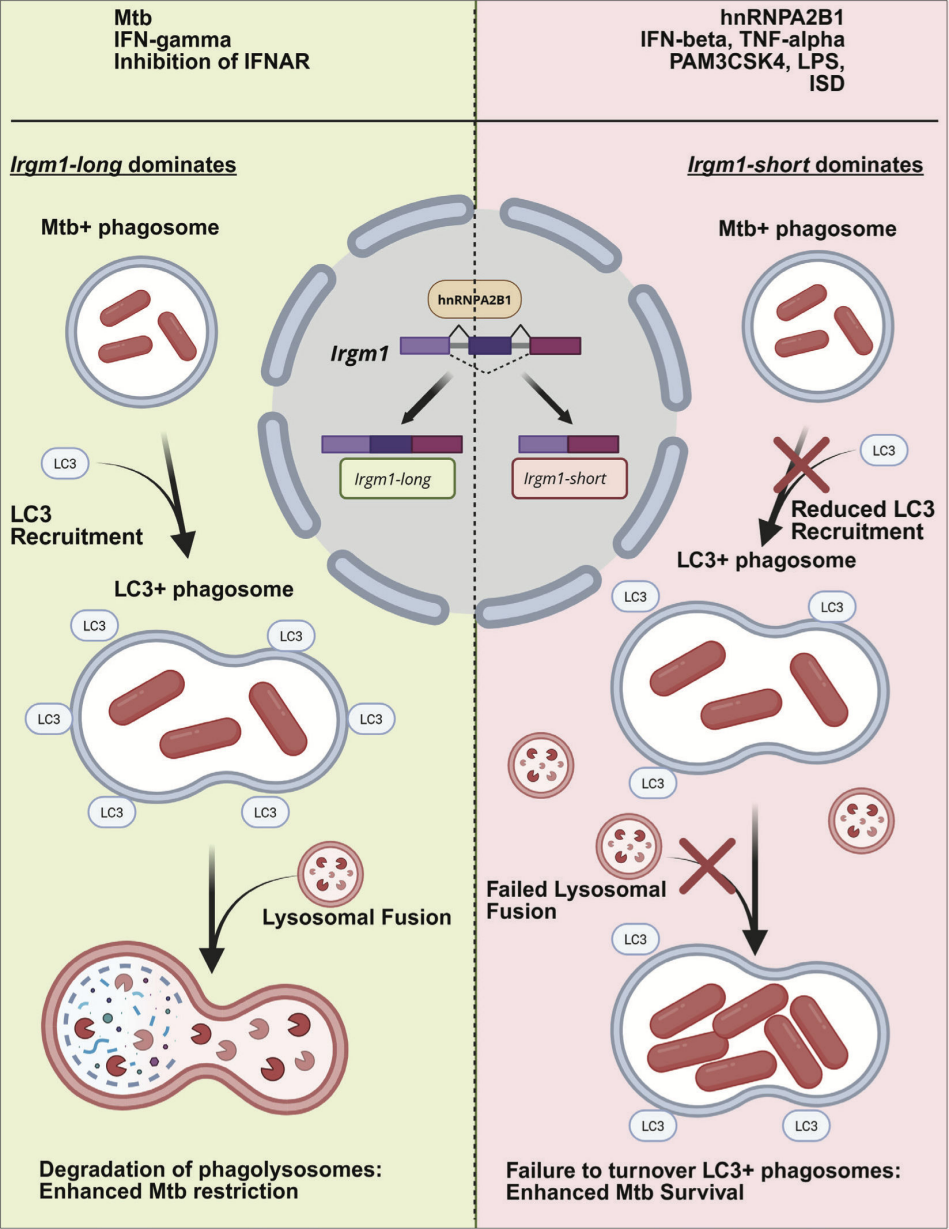
Model: Irgm1 isoforms differentially impact autophagic clearance of Mtb In stimulated macrophages, *Irgm1* transcripts increase in abundance and are alternatively spliced into long or short isoforms by hnRNPA2B1 (with the presence of hnRNPA2B1 favoring skipping of exon 2 and generation of the short isoform). The decision to make Irgm1-short vs. Irgm1-long can be influenced by different immune stimuli, with Mtb infection, IFN-γ, and IFNAR blocking promoting Irgm1-long, and PRR stimulation (cGAS-STING, TLR1/2/6) and cytokine treatment (TNF-α, IFN-β) eliciting more balanced expression of Irgm1-long and -short. Our data suggest that Irgm1-long supports autophagy, enabling LC3 recruitment to Mtb-containing phagosomes and subsequent lysosomal fusion, thereby restricting bacterial replication. In contrast, Irgm1-short impairs both LC3 and lysosomal recruitment, resulting in failure to control intracellular Mtb. Created in BioRender. Chapman, M. (2026) https://BioRender.com/3ldv1ks

Although differential subcellular localization of Irgm1-long and short has been previously reported (73), our data shed new light on how these proteins may function differently during bacterial infection of macrophages. Most notably, we show that expression of Irgm1-long promotes restriction of Mtb replication in macrophages, while expression of Irgm1-short creates a more permissive niche for Mtb (Fig. 5B). The mechanisms underlying these phenotypes appear to be somewhat complex. Unlike Irgm1-long, expression of Irgm1-short is not sufficient to promote LC3+ recruitment to Mtb bacilli (Fig. 5C and D). It is, however, sufficient to inhibit lysosomal function, as evidenced by the lack of colocalization between *E. coli* and the pH-sensitive dye LysoTracker (Fig. 5E through G). Lysosomal defects have previously been reported in mouse embryonic fibroblasts lacking Irgm1 (77). Such defects are attributed to mislocalization of GKS proteins (e.g., Irga6, Irgb6, and Irgb10) to lysosomes and subsequent lysosomal dysfunction (evidenced by impaired dequenching of the pH-sensitive fluorescent dye DQ-BSA) (77). Although we cannot distinguish whether Irgm1-short inhibits lysosome acidification, fusion of lysosomes with internalized *E. coli*, or both, our data suggest that Irgm1-short expression generally phenocopies loss of the *Irgm1* gene locus. These findings support a model whereby Irgm1-short opposes the function of Irgm1-long, acting as an internal rheostat to tune lysosomal capacity in response to infection or other external cues. Given that AS impacts the inclusion of a N-terminal targeting region, yet leaves the GTPase domain intact, this differential subcellular localization may sequester Irgm1 binding partners away from compartments of the cell where they are needed to function in cellular processes like autophagy (Fig. 7).

It is not entirely clear why we see more LC3+ Mtb bacilli in Irgm1-long vs. Irgm1-short cells. Although our finding is consistent with Irgm1 knockdown cells harboring fewer LC3+ BCG (83), we do not suspect this occurs through direct association of Irgm with the Mtb phagosome. Early reports of Irgm1 recruitment to Mtb phagosomes (93) have largely been dismissed (73) and we did not detect any evidence for either Irgm1-long or -short colocalizing with Mtb (data not shown). We propose that instead of Irgm1 physically directing LC3+ to Mtb phagosomes, its loss/overexpression dysregulates components of the autophagy machinery. The Deretic lab has shown that IRGM1 interacts with core components of the autophagy machinery and IRGM1 knockdown decreases steady state levels of pULK-1, ATG5, and ATG16L1 in U937 cells (94). Overexpression of Irgm1-long, but not Irgm1-short, may increase the stability or availability of one or more of these factors, such that they are ready to participate in antimycobacterial selective autophagy. Altered lysosomal function may also influence the number of LC3+ bacilli in Irgm1-long vs. -short expressing cells, by misregulating autophagolysosome formation or turnover of targeted cargo.

Our finding that *Irgm1* AS is regulated by distinct immune agonists hints at the potential for hnRNPA2B1 itself to be regulated. Various stressors (e.g., heat shock and genotoxic stress) are known to alter the phosphorylation status and subcellular localization of SR/hnRNP family members to control AS decisions (95–98). Analogous regulation has been reported in macrophages responding to immune agonists and/or infection (99, 100). Our own data demonstrate that LPS-directed p38-dependent phosphorylation of hnRNP M at S574 alters its capacity to associate with the IL6 genomic locus and repress IL6 maturation (12). We propose that post-translational modification of A2B1, downstream of specific signals, toggles AS of Irgm1. High-throughput proteomics studies have identified many PTM sites on hnRNPA2B1 (Fig. S5F) (101) and quantitative proteomics of Mtb-infected BMDMs hint at differential phosphorylation of A2B1 at Y244 (102). The outstanding question, then, is what signals could direct this modification and others? We found that Mtb and IFN-γ selectively promote splicing of Irgm1-long (Fig. 6A and H). In the case of Mtb, this required an intact ESX-1 secretion system, suggesting cytosolic access, the detection of Mtb-derived PAMPs like dsDNA/dsRNA, and/or secreted virulence proteins are somehow integrated to signal A2B1 to make more Irgm1-long relative to -short. It is notable that the only cytokine (of those tested) that promoted the generation of Irgm1-long was IFN-γ, a well-established inducer of IRGM1-dependent antimycobacterial immunity. Despite sharing many signaling components, IFN-γ is seemingly better than IFN-β at promoting Irgm1-long, suggesting that signaling downstream of IFNGR may deliver a unique signal to A2B1. Molecular dissection of these signaling cascades via genetics and inhibitors will provide new insights into how macrophages differentially regulate AS to generate a specialized antibacterial proteome.

Our finding that A2B1 is required to constrain Mtb replication (Fig. 2B) positions this RNA-binding protein as a *bona fide* mycobacterial restriction factor, but the mechanisms underlying this phenotype remain unclear. If Irgm1 AS were the “culprit” of the permissive phenotype in A2B1 KO BMDMs, then we would expect the short, permissive isoform of Irgm1 to dominate in these cells. That is, in fact, the opposite of what we see (Fig. 4), suggesting that one or more other AS events in A2B1 KO BMDMs—or perhaps other A2B1-linked phenotypes (miRNA biogenesis? m6A levels?) are responsible for this phenotype. Future experiments applying the framework employed here to other A2B1-sensitive AS isoforms will be needed to explain why loss of A2B1 promotes Mtb replication.

## MATERIALS AND METHODS

### Mice

C57BL/6J (Jackson Laboratories, stock no. 00064) and hnRNPA2B1-KO mice (a gift from the Carpenter laboratory, University of California, Santa Cruz) were bred and maintained under approved protocols of the Texas A&M College of Medicine and Vanderbilt University Medical Center’s Institutional Animal Care and Use Committees. Age- and sex-matched littermates (8–16 weeks) were used for all experiments. Mice were housed on a 12 h light/dark cycle with *ad libitum* access to food and water.

### Cell culture

Immortalized bone-marrow-derived macrophages (iBMDMs) and HEK293T cells were cultured in high-glucose DMEM (Thermo Fisher) supplemented with 10% FBS (Millipore) and 0.2% HEPES at 37°C in 5% CO₂. All cell lines tested negative for mycoplasma contamination.

### Generation of bone marrow-derived macrophages

Primary BMDMs were differentiated from femurs and tibias of 8- to 12-week-old C57BL/6J mice as previously described (103). Briefly, marrow progenitors were cultured for 6 days in DMEM containing 10% heat-inactivated FBS and 20% L929-conditioned M-CSF. Cells were harvested in PBS–EDTA, washed, and replated for infection or stimulation assays.

### Immortalization and stable expression lines

WT BMDMs were immortalized by J2 CRE retroviral transduction to generate iBMDMs (29). Stable RAW 264.7 and iBMDM lines expressing GFP-FLAG, Irgm1-short-FLAG, or Irgm1-long-FLAG were produced using pLenti PGK-Puro DEST vectors followed by puromycin selection. Expression was confirmed by anti-FLAG immunoblotting.

### Mtb infections

All pathogen work was conducted under appropriate biosafety conditions. *Mycobacterium tuberculosis* Erdman and LuxABCDE-Erdman strains were grown in Middlebrook ^7^H9 broth with OADC, Tween-80, and glycerol. BMDMs were infected at an MOI of 1, centrifuged (1,000 RPM for 10 min, VWR MegaStar 4.0) briefly to synchronize infection, and washed 2 h later. CFU counts were obtained by serial dilution on 7H10 agar; luminescence (RLU) was measured on a TECAN Spark reader.

### Innate immune stimulations

BMDMs or RAW cells were stimulated for 4 h with 100 ng/mL LPS (InvivoGen) or 1 µg/mL Pam3CSK4 (InvivoGen) or transfected with 1 µg/mL interferon-stimulating DNA (ISD) (Patrick Lab) or poly(I:C) (InvivoGen) using Lipofectamine. Cytokine stimulations used 50 IU/mL IFN-β (PBL Assay Science), 50 IU/mL IFN-γ (PBL Assay Science), or 20 ng/mL TNF-α (PeproTech). Inhibition of innate immune pathways was performed by incubating cells in 5 µM H-151 (STING) (Cayman) or 160 IU/mL MAR1-5A3 (eBioscience) (Thermo Fisher) for 5 h prior to infection, then leaving the inhibitor in the culture for the remainder of the infection.

### RNA isolation and sequencing

RNA was extracted in TRIzol and purified with Direct-zol RNA Miniprep kits (Zymo Research), including on-column DNase digestion. Library preparation and sequencing (paired-end 150 bp, NovaSeq 6000) were performed by the Baylor College of Medicine Genomic and RNA Profiling Core (GARP) Core. Reads were trimmed with fastp (v 1.0.1) (104) and aligned with STAR (v 2.7.11b) (105) to the GRCm39/Gencode v44 reference. Differential expression was analyzed with featureCounts (v2.1.1) (106) and DESeq2 (v1.46.0)(107), and alternative splicing was evaluated using MAJIQ and VOILA (v 2.5.11; |ΔPSI| ≥ 0.15, *P* ≥ 0.9) (56, 108). Protein-coding status was determined by IsoformSwitchAnalyzeR (v) (109) to screen isoforms for the longest ORF; CPAT (v 3.0.5) (110) was then used to remove ORFs with low probabilities of being translated (< 0.5). The gene locus was considered protein-coding if at least one isoform met these criteria. DeepLoc2.1 (74) was used to determine subcellular localization. Gene-ontology was performed using ClusterProfiler (111), ORAs referenced the GO: Biological Process database (112), GSEAs referenced the HALLMARK database (113, 114). Predicted structures for isoforms were obtained from the AlphaFold Protein Structure Database (115, 116), and .pdb files were visualized for annotation using the Pymol software (v2.5.4) (117). Plots and statistical analyses were generated in R (v 4.4) using ggplot2 (118, 119).

### Quantitative RT-PCR and semi-quantitative PCR

DNase-treated RNA was reverse-transcribed with iScript cDNA Synthesis Kit (Bio-Rad). qPCR was performed with PowerUp SYBR Green Master Mix (Thermo Fisher) on a QuantStudio Flex6 instrument. For gel-based analysis, Q5 polymerase (NEB) amplicons were resolved on 2% agarose and visualized on a Li-COR Odyssey imager.

### Immunoblotting

Cells were lysed in 1% SDS, boiled, and analyzed by SDS-PAGE (4%–20% gradient) and transferred to 0.2 µM PVDF membranes (Immobilon-PSQ Millipore Sigma). Primary antibodies included anti-FLAG (FG4R Invitrogen, 1:1,000), anti-IRGM (E6P7W Cell Signaling Technology, 1:1,000), anti-HNRNPA2B1 (3H6F7 ProteinTech, 1:1,000), and anti-β-actin (ab8226 Abcam, 1:1,000). Secondary antibodies included Goat-anti-mouse IgG-800CW (926-32210 Licor, 1:10,000) and Goat-anti-rabbit IgG-680RD (926-68071 Licor, 1:10,000).

### Immunofluorescence microscopy

For immunofluorescence, cells on coverslips were fixed (4% PFA), permeabilized (0.2% Triton X-100), stained with primary and fluorophore-conjugated secondary antibodies, and mounted in ProLong Diamond. Fluorescence microscopy was performed using Zeiss LSM880 Airyscan and LSM710 confocal microscopes through the Vanderbilt Cell Imaging Shared Resource. Primary antibodies included anti-FLAG (FG4R Invitrogen, 1:500), anti-LC3 (L10382 Thermofisher, 1:500), and anti-LAMP1 (E5N9Z Cell Signaling Technology, 1:500).

### Image processing and segmentation

Cells were segmented using DIC/BF images and Cellpose (cyto2 model) with standard preprocessing and small-object filtering. Mtb bacilli were identified from the Mtb fluorescence channel by intensity-based thresholding and morphological cleanup.

### Fluorescence quantification, colocalization, and statistics

Recruitment of LC3 and LAMP1 to Mtb was quantified by measuring mean fluorescence intensity within a thin ring surrounding each segmented bacillus, as well as within whole-cell cytoplasmic masks. Bacilli positive for each marker were defined using a uniform intensity threshold applied across conditions. Colocalization between markers and Mtb was assessed using Manders’ (M1/M2) and Pearson correlation coefficients computed from background-subtracted pixel intensities within cytoplasmic or bacillus-associated regions. All statistical analyses were performed in Python using SciPy and custom permutation tests. Group comparisons used appropriate parametric or nonparametric tests with Benjamini–Hochberg correction where indicated. At least 300 cells per condition were analyzed, and error bars represent SD unless noted otherwise.

### Live-cell imaging and analysis

Cells were incubated in 100 nm lysotracker-deepred (L12492 Thermofisher), and 1 µg/mL Hoechst stain (H21486 Thermofisher) for 30 min then infected with *E. coli* stably expressing mCherry at an MOI = 10 and centrifuged (1,000 RPM for 10 min, VWR MegaStar 4.0) briefly to synchronize infection. Media were replaced with optical complete DMEM supplemented with 1 µg/mL Hoechst stain. Time-lapse CZI files were acquired on a Zeiss LSM880 Airyscan microscope (four channels: LysoTracker, DIC, DAPI, mCherry) using identical settings (0.215-µm pixels; 2-min intervals; 1 h duration). Images were Z-projected and segmented in Cellpose to identify nuclei and cytoplasm. For each cell and time point, LysoTracker and mCherry mean intensities and Pearson/Manders colocalization coefficients were quantified. Cells were tracked by nearest-centroid linking (5-µm maximum displacement), and per-cell metrics were aggregated across movies to generate genotype-specific lysosomal recruitment and bacterial colocalization dynamics.

### Functional assays

Cells (2.5 × 10⁴/mL) were spin-infected (10 min, 1,000 rpm; MOI 10), washed once with PBS, and overlaid with 0.1% PI-containing medium. PI fluorescence was measured on a TECAN Spark plate reader. Triton X-100 (0.1%) wells served as genotype-matched positive controls and media-only wells as background. Percent cell death was calculated as:


$$Cell\;death\;\left(\%\right)=\frac{well\;value-background}{TritonX\;mean-background}\times 100$$

### Statistical analysis and figure generation

All statistical analyses were performed in GraphPad Prism (v 10.4.1). Two-tailed unpaired Student’s *t*-tests or Mann-Whitney tests were applied as appropriate. Data are mean ± SEM from ≥3 biological replicates. Our model was made with BioRender (RI293O6ST8).

## Data Availability

RNA-seq data supporting this study are available from the Gene Expression Omnibus (GEO accession GSE313377). Additional data and reagents are available from the corresponding authors upon request.

## SUPPLEMENTAL MATERIAL

Supplemental figures iai.00732-25-s0001.pdf Fig. S1 to S5.

Supplemental tables iai.00732-25-s0002.xls Tables S1 to S3.

Video S1 iai.00732-25-s0003.avi Live-cell imaging of LysoTracker and mCherry *E. coli* in GFP-, Irgm1-long-, and Irgm1-short-expressing iBMDMs.

ASM does not own the copyrights to Supplemental Material that may be linked to, or accessed through, an article. The authors have granted ASM a non-exclusive, world-wide license to publish the Supplemental Material files. Please contact the corresponding author directly for reuse.
