# Supplementary material for: HnRNPA2B1 tunes antimycobacterial immune responses in macrophages through alternative splicing of Irgm1
Source: Infect Immun. 2026 Jun 18;94(7):e00732-25. doi: 10.1128/iai.00732-25 (PMC13367058; doi:10.1128/iai.00732-25)
Supplement: Supplemental figures — Fig. S1 to S5. [file iai.00732-25-s0001.pdf]

Chapman et al.

Supplemental Figure Legends

Figure S1.

(A) Pie chart of all LSVs from WT Mtb infection, agnostic of DEG category.

(B) List of AS genes with dPSI > 0.30, sorted by LSV category.

(C) ORA of DEGs during Mtb infection in WT BMDMs, top 15 terms

(D) ORA of AS genes during Mtb infection in WT BMDMs, top 15 terms

Statistical tests: RNA-seq differential expression (C) was performed using DESeq2 with Wald test statistics and Benjamini–Hochberg correction; genes were considered differentially expressed at adjusted $P < 0.05$. Alternative splicing events (A, B, D) were identified using MAJIQ with a threshold of $\Delta PSI > 0.15$ and probability > 0.90 unless otherwise indicated. Over-representation analysis (C–D) was performed using a hypergeometric test with false discovery rate correction. Data are derived from $n = 3$ biologically independent samples unless otherwise noted.

Figure S2.

(A) Propidium Iodide incorporation assay measuring cell death in WT or hnRNPA2B1-KO BMDMs during Mtb infection over 24 hours. n=6.
(B) Gene set enrichment curves from most downregulated pathways in hnRNPA2B1-KO BMDMs during Mtb infection, from GSEA HALLMARK terms; Inflammatory Gene Response
(C) As in (B); TNF-α Signaling via NFκB
(D) As in (B); Hypoxia
(E) Gene set enrichment curves from most upregulated pathways in hnRNPA2B1-KO BMDMs during Mtb infection, from GSEA HALLMARK terms: Mitotic Spindle
(F) As in (E); G2M Checkpoint
(G) As in (E); Interferon Alpha Response
(H) RT-qPCR of selected DEGs in WT and hnRNPA2B1-KO BMDMs post-Mtb infection.

Statistical tests: Data are presented as mean ± SD from $n = 6$ biologically independent samples for cell death assays (A) and $n = 3$ biologically independent samples for RT-qPCR (H), unless otherwise noted. Propidium iodide incorporation assays (A) were analyzed using two-way ANOVA with repeated measures and multiple comparisons correction. RT-qPCR experiments (H) were analyzed using two-way ANOVA with multiple comparisons correction. Gene set enrichment analysis (B–G) was performed using GSEA with significance determined by false discovery rate (FDR < 0.25). $P < 0.05$, *$P < 0.01$, **$P < 0.001$, ***$P < 0.0001$.

<u>Figure S3</u>.

(A) COBALT amino acid alignment of human IRGM isoforms, murine Irgm1 isoforms, Irgm2 isoforms, and Irgm3. High-confidence region of homology is annotated in red along with Interpro domain calling. NCBI IDs are listed.

(B) Diagram describing primer design for semi-quantitative RT-PCR of *Irgm1.*

(C) Diagram describing primer design for RT-qPCR of *Irgm1.*

(D) Western blot of IRGM abundance in WT and hnRNPA2B1-KO BMDMs post-Mtb infection.

(E) Western blot of ectopically expressed N-terminal 3xFLAG-hnRNPA2B1 or GFP in iBMDMs.

(F) DeepLoc2.1 subcellular localization prediction of *Irgm1* isoform amino acid sequences.

(G) Predicted lysosomal targeting dileucine motif in the N-terminus of Irgm1-long

(H) Western blot of ectopically expressed N-FLAG-Irgm1-isoforms or GFP control in HEK293T cells.

(I) Immunofluorescence microscopy of 3xFLAG-Irgm1-long, 3xFLAG-Irgm1-short, or 3xFLAG-GFP subcellular localization (FLAG, green) with DAPI (blue) counter stain in HEK293T cells.

Statistical tests: No statistical tests were performed for panels A–H unless otherwise indicated. Protein alignments (A) were generated using COBALT, and subcellular localization predictions (F) were performed using DeepLoc 2.1. Immunoblots (D, E, G) and immunofluorescence images (H) are representative of independent experiments.

Figure S4.

(A) Propidium iodide incorporation assay measuring cell death in GFP, Irgm1-long, and Irgm1-short-expressing iBMDMs during Mtb infection over 24 hours. n=6.

(B) Brightfield imaging of monolayers from GFP, Irgm1-long, or Irgm1-short-expressing iBMDMs 0-, 3-, and 5-days post-Mtb infection (from **Fig. 5B**)

(C) Quantification of **Fig. 5D**; total Mtb area per field. n=6

(D) Quantification of **Fig. 5D**; LC3 MFI per cell. n=6

(E) Total LAMP1 MFI per field. n=6

(F) % LAMP1+ bacilli per field. n=6

(G) Mean fluorescence intensity of anti-FLAG in Irgm1-long and -short iBMDMs at baseline

(H) Live-cell fluorescence microscopy of LysoTracker (green) and nucleus (blue) prior to *E. coli* infection in GFP, Irgm1-long, and Irgm1-short iBMDMs.

(I) Quantification of (G); LysoTracker MFI per cell.

Statistical tests: Data are presented as mean ± SD from $n = 6$ biologically independent samples unless otherwise noted. Propidium iodide incorporation assays (A) were analyzed using two-way ANOVA with repeated measures and multiple comparisons correction. Quantification of imaging-based assays (C–F, I) was performed using one-way ANOVA with multiple comparisons correction. Mean fluorescence intensity of FLAG-tagged proteins at baseline (G) was analyzed using one-way ANOVA. Representative brightfield and fluorescence images (B, H) are shown from independent experiments. $P < 0.05$, *$P < 0.01$, **$P < 0.001$, ***$P < 0.0001$.

<u>Figure S5</u>.

(A) Semiquantitative RT-PCR of *Irgm1* exon 2 inclusion in WT and hnRNPA2B1-KO BMDMs pre-treated with H-151 (5 μM) post-Mtb infection (MOI = 5). n=3. Quantification on right represented as band intensity of *Irgm1-long* (top) over band intensity of *Irgm1-short* (bottom).

(B) As in A, but pre-treatment with MAR1-5A3 (160 IU/mL)

(C) As in A, but no pre-treatment

(D) RT-qPCR of *Rsad2* in BMDMs with or without H-151 or MAR1-5A3 pre-treatment during Mtb infection

(E) Reported post-translational modifications on murine hnRNPA2B1 depicted on PhosphoSitePlus.

Data are presented as mean ± SD from *n* = 3 biologically independent samples unless otherwise noted. Semiquantitative RT-PCR band intensity quantification (A–C) was expressed as the ratio of Irgm1-long to Irgm1-short and analyzed using two-way ANOVA with multiple comparisons correction for treatment/genotype and time. RT-qPCR experiments (D) were analyzed using two-way ANOVA with multiple comparisons correction. Post-translational modification annotations (F) were curated from PhosphoSitePlus. $P < 0.05$, *$P < 0.01$, **$P < 0.001$, ***$P < 0.0001$.

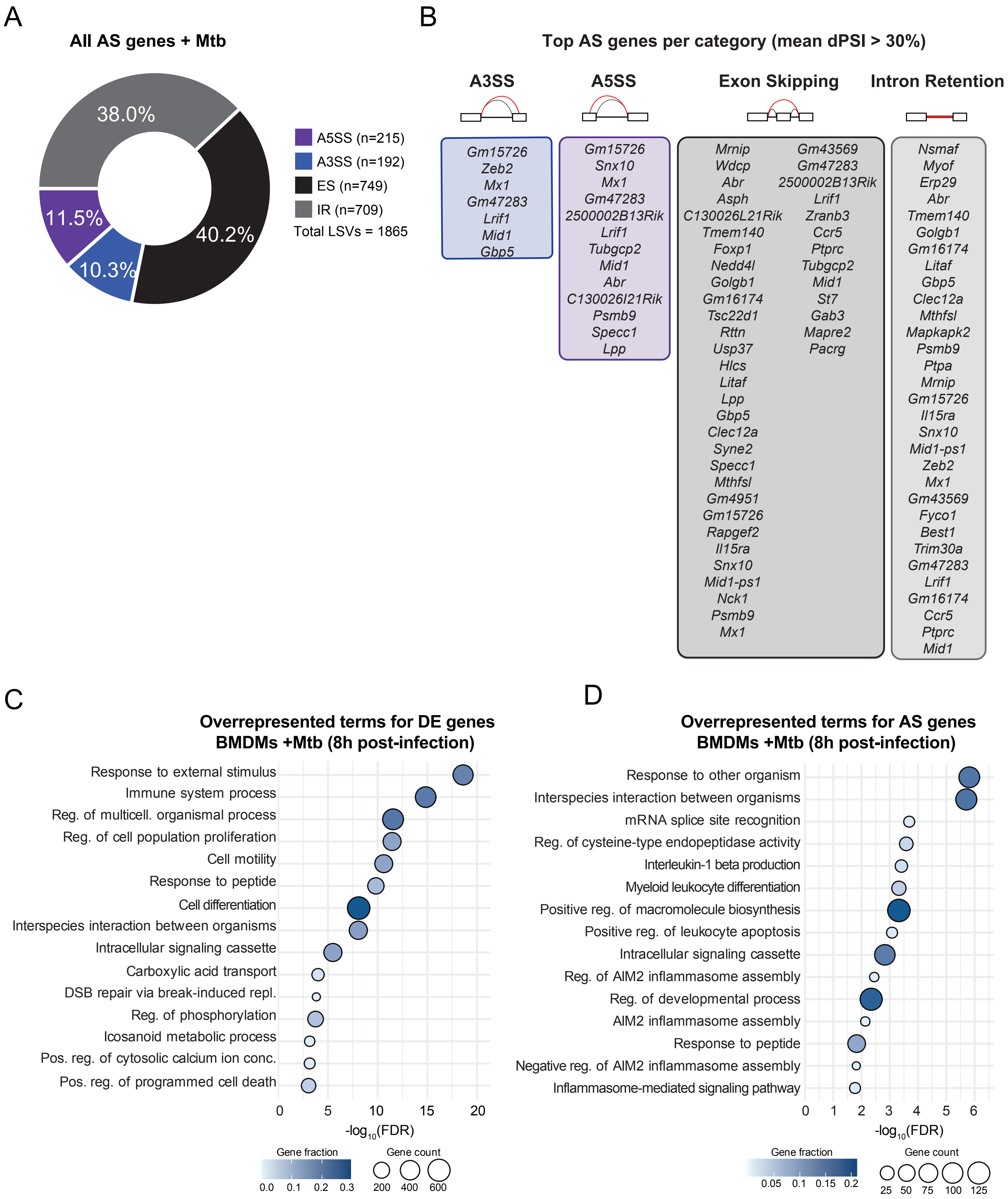
A
All AS genes + Mtb
38.0%
40.2%
11.5%
10.3%
A5SS (n=215)
A3SS (n=192)
ES (n=749)
IR (n=709)
Total LSVs = 1865
B
Top AS genes per category (mean dPSI > 30%)
A3SS
Gm15726
Zeb2
Mx1
Gm47283
Lrif1
Mid1
Gbp5
A5SS
Gm15726
Snx10
Mx1
Gm47283
2500002B13Rik
Lrif1
Tubgcp2
Mid1
Abr
C130026I21Rik
Psmb9
Specc1
Lpp
Exon Skipping
Mrnip
Wdcp
Abr
Asph
C130026L21Rik
Tmem140
Foxp1
Nedd4l
Golgb1
Gm16174
Tsc22d1
Rttn
Usp37
Hlcs
Litaf
Lpp
Gbp5
Clec12a
Syne2
Specc1
Mthfsl
Gm4951
Gm15726
Rapgef2
Il15ra
Snx10
Mid1-ps1
Nck1
Psmb9
Mx1
Gm43569
Gm47283
2500002B13Rik
Lrif1
Zranb3
Ccr5
Ptprc
Tubgcp2
Mid1
St7
Gab3
Mapre2
Pacrg
Intron Retention
Nsmaf
Myof
Erp29
Abr
Tmem140
Golgb1
Gm16174
Litaf
Gbp5
Clec12a
Mthfsl
Mapkapk2
Psmb9
Ptpa
Mrnip
Gm15726
Il15ra
Snx10
Mid1-ps1
Zeb2
Mx1
Gm43569
Fyco1
Best1
Trim30a
Gm47283
Lrif1
Gm16174
Ccr5
Ptprc
Mid1
C
Overrepresented terms for DE genes
BMDMs +Mtb (8h post-infection)
Response to external stimulus
Immune system process
Reg. of multicell. organismal process
Reg. of cell population proliferation
Cell motility
Response to peptide
Cell differentiation
Interspecies interaction between organisms
Intracellular signaling cassette
Carboxylic acid transport
DSB repair via break-induced repl.
Reg. of phosphorylation
Icosanoid metabolic process
Pos. reg. of cytosolic calcium ion conc.
Pos. reg. of programmed cell death
0 5 10 15 20
-log10(FDR)
Gene fraction
0.0 0.1 0.2 0.3
Gene count
200 400 600
D
Overrepresented terms for AS genes
BMDMs +Mtb (8h post-infection)
Response to other organism
Interspecies interaction between organisms
mRNA splice site recognition
Reg. of cysteine-type endopeptidase activity
Interleukin-1 beta production
Myeloid leukocyte differentiation
Positive reg. of macromolecule biosynthesis
Positive reg. of leukocyte apoptosis
Intracellular signaling cassette
Reg. of AIM2 inflammasome assembly
Reg. of developmental process
AIM2 inflammasome assembly
Response to peptide
Negative reg. of AIM2 inflammasome assembly
Inflammasome-mediated signaling pathway
0 1 2 3 4 5 6
-log10(FDR)
Gene fraction
0.05 0.1 0.15 0.2
Gene count
25 50 75 100 125

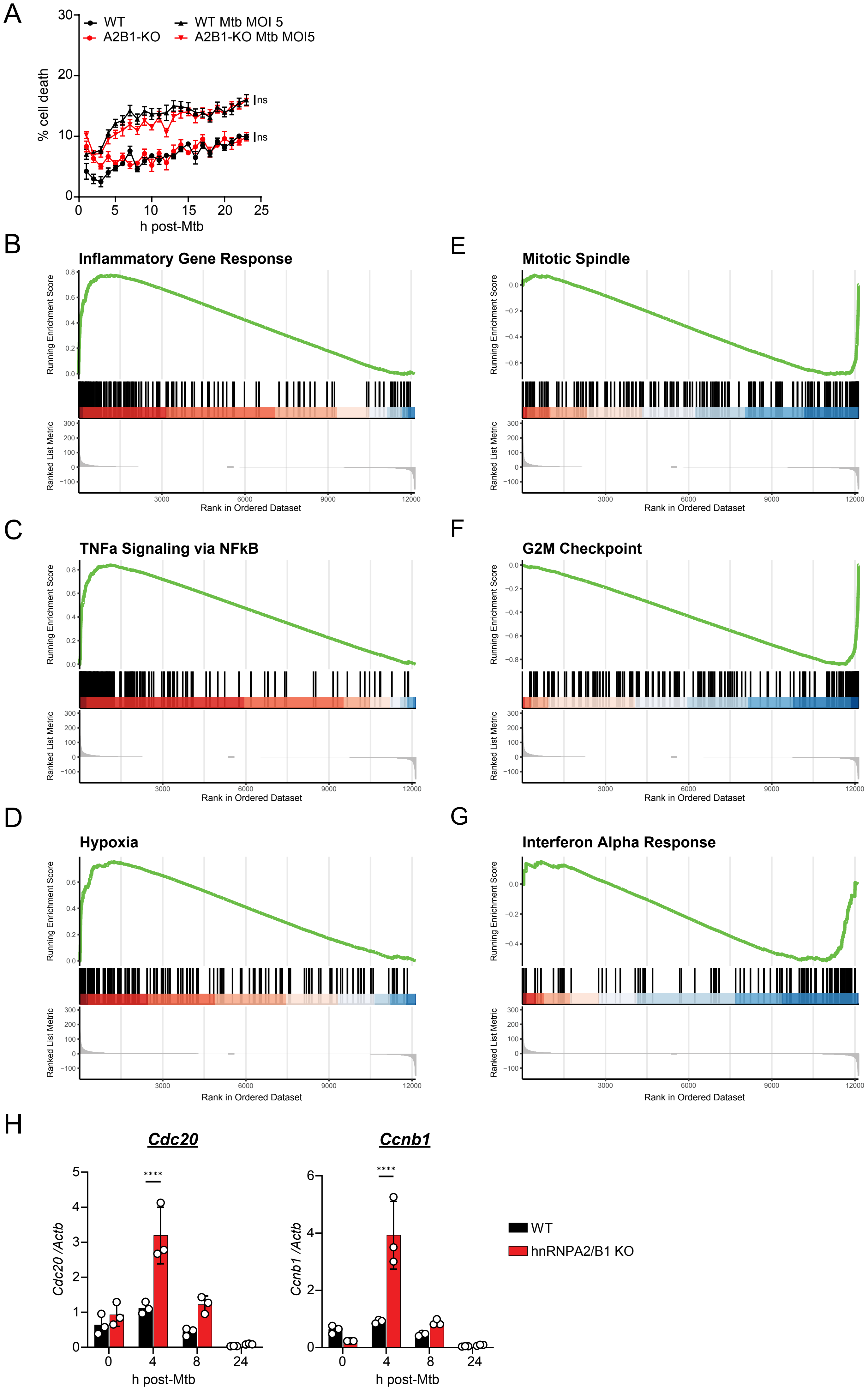
A
WT
A2B1-KO
WT Mtb MOI 5
A2B1-KO Mtb MOI5
% cell death
h post-Mtb
ns
ns
B
Inflammatory Gene Response
C
TNFa Signaling via NFkB
D
Hypoxia
E
Mitotic Spindle
F
G2M Checkpoint
G
Interferon Alpha Response
Running Enrichment Score
Ranked List Metric
Rank in Ordered Dataset
H
Cdc20
Ccnb1
Cdc20 /Actb
Ccnb1 /Actb
****
h post-Mtb
WT
hnRNPA2/B1 KO

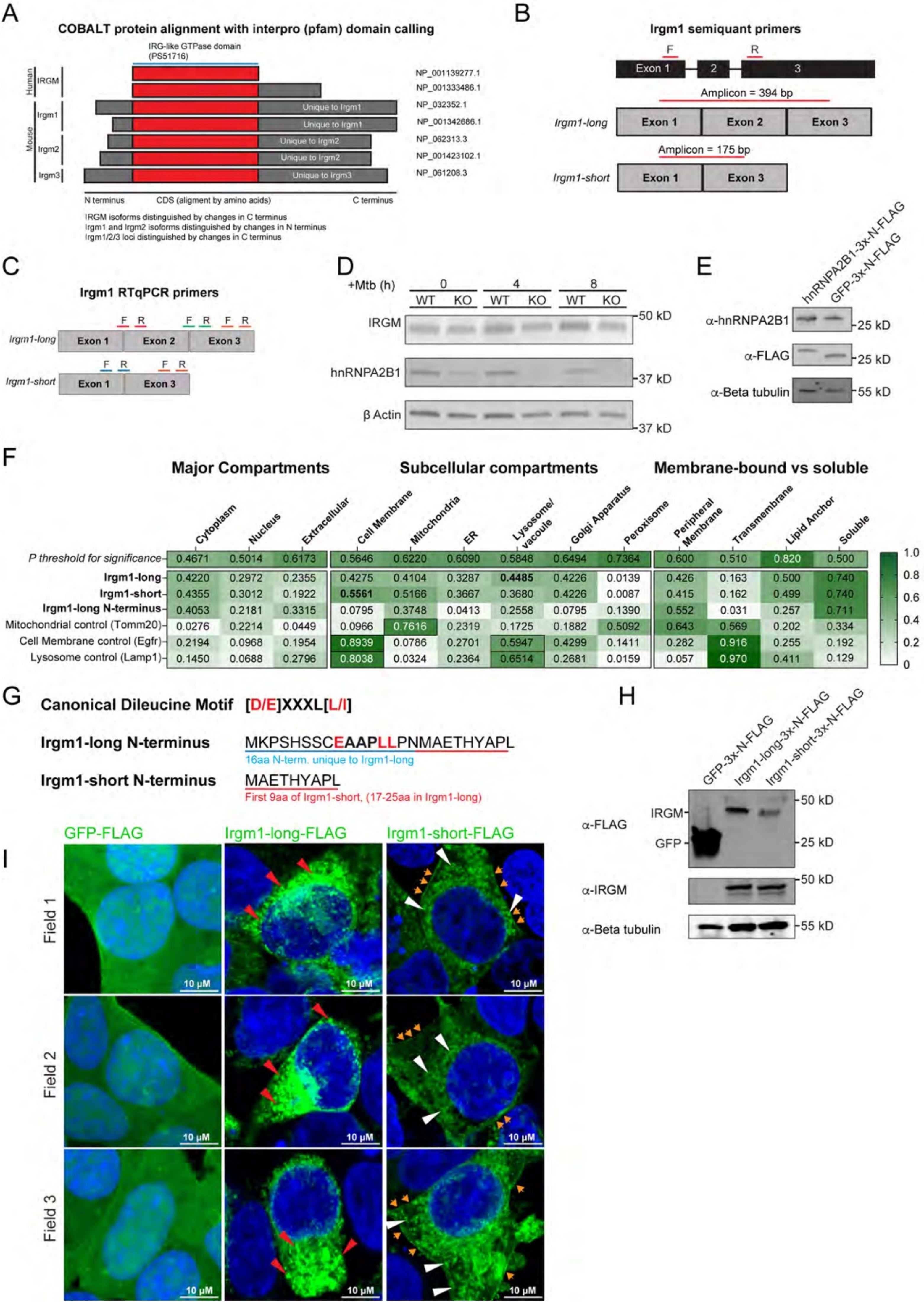
A
COBALT protein alignment with interpro (pfam) domain calling
IRG-like GTPase domain (PS51716)
Human
IRGM
Mouse
Irgm1
Irgm2
Irgm3
Unique to Irgm1
Unique to Irgm2
Unique to Irgm3
NP_001139277.1
NP_001333486.1
NP_032352.1
NP_001342686.1
NP_062313.3
NP_001423102.1
NP_061208.3
N terminus
CDS (aligment by amino acids)
C terminus
IRGM isoforms distinguished by changes in C terminus
Irgm1 and Irgm2 isoforms distinguished by changes in N terminus
Irgm1/2/3 loci distinguished by changes in C terminus
B
Irgm1 semiquant primers
F
R
Exon 1
2
3
Amplicon = 394 bp
Irgm1-long
Exon 1
Exon 2
Exon 3
Amplicon = 175 bp
Irgm1-short
Exon 1
Exon 3
C
Irgm1 RTqPCR primers
Irgm1-long
Irgm1-short
D
+Mtb (h)
0
4
8
WT
KO
IRGM
hnRNPA2B1
β Actin
50 kD
37 kD
E
hnRNPA2B1-3x-N-FLAG
GFP-3x-N-FLAG
α-hnRNPA2B1
α-FLAG
α-Beta tubulin
25 kD
55 kD
F
Major Compartments
Subcellular compartments
Membrane-bound vs soluble
Cytoplasm | Nucleus | Extracellular | Cell Membrane | Mitochondria | ER | Lysosome/vacoule | Golgi Apparatus | Peroxisome | Peripheral Membrane | Transmembrane | Lipid Anchor | Soluble
P threshold for significance | 0.4671 | 0.5014 | 0.6173 | 0.5646 | 0.6220 | 0.6090 | 0.5848 | 0.6494 | 0.7364 | 0.600 | 0.510 | 0.820 | 0.500
Irgm1-long | 0.4220 | 0.2972 | 0.2355 | 0.4275 | 0.4104 | 0.3287 | 0.4485 | 0.4226 | 0.0139 | 0.426 | 0.163 | 0.500 | 0.740
Irgm1-short | 0.4355 | 0.3012 | 0.1922 | 0.5561 | 0.5166 | 0.3667 | 0.3680 | 0.4226 | 0.0087 | 0.415 | 0.162 | 0.499 | 0.740
Irgm1-long N-terminus | 0.4053 | 0.2181 | 0.3315 | 0.0795 | 0.3748 | 0.0413 | 0.2558 | 0.0795 | 0.1390 | 0.552 | 0.031 | 0.257 | 0.711
Mitochondrial control (Tomm20) | 0.0276 | 0.2214 | 0.0449 | 0.0966 | 0.7616 | 0.2319 | 0.1725 | 0.1882 | 0.5092 | 0.643 | 0.569 | 0.202 | 0.334
Cell Membrane control (Egfr) | 0.2194 | 0.0968 | 0.1954 | 0.8939 | 0.0786 | 0.2701 | 0.5947 | 0.4299 | 0.1411 | 0.282 | 0.916 | 0.255 | 0.192
Lysosome control (Lamp1) | 0.1450 | 0.0688 | 0.2796 | 0.8038 | 0.0324 | 0.2364 | 0.6514 | 0.2681 | 0.0159 | 0.057 | 0.970 | 0.411 | 0.129
1.0
0.8
0.6
0.4
0.2
0
G
Canonical Dileucine Motif [D/E]XXXL[L/I]
Irgm1-long N-terminus MKPSHSSCEAAPLLPNMAETHYAPL
16aa N-term. unique to Irgm1-long
Irgm1-short N-terminus MAETHYAPL
First 9aa of Irgm1-short, (17-25aa in Irgm1-long)
H
GFP-3x-N-FLAG
Irgm1-long-3x-N-FLAG
Irgm1-short-3x-N-FLAG
α-FLAG
IRGM
GFP
α-IRGM
α-Beta tubulin
50 kD
25 kD
55 kD
I
GFP-FLAG
Irgm1-long-FLAG
Irgm1-short-FLAG
Field 1
Field 2
Field 3
10 μM

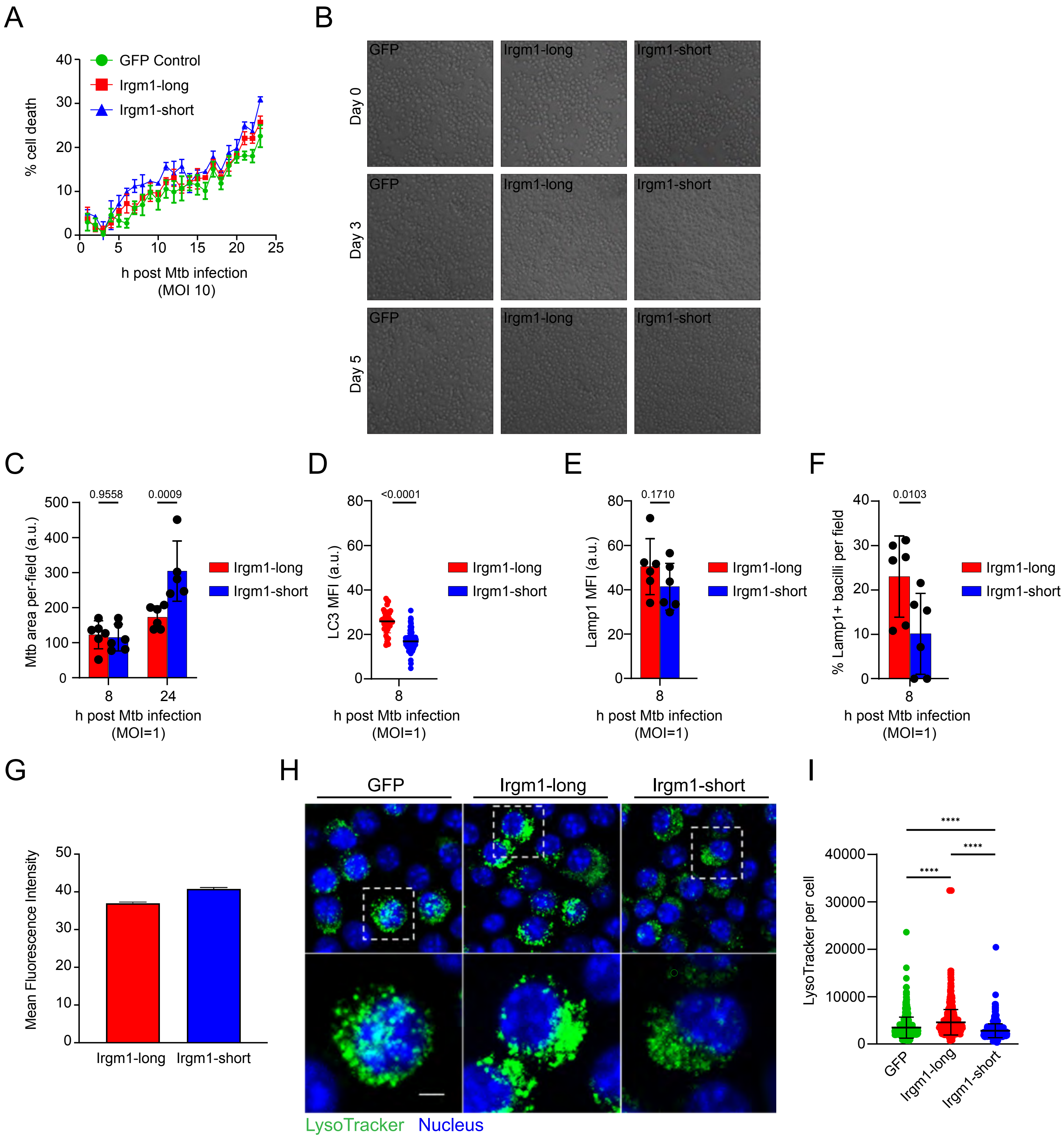

A
% cell death
GFP Control
Irgm1-long
Irgm1-short
h post Mtb infection
(MOI 10)
B
GFP
Irgm1-long
Irgm1-short
Day 0
Day 3
Day 5
C
Mtb area per-field (a.u.)
0.9558
0.0009
h post Mtb infection
(MOI=1)
D
LC3 MFI (a.u.)
<0.0001
E
Lamp1 MFI (a.u.)
0.1710
F
% Lamp1+ bacilli per field
0.0103
G
Mean Fluorescence Intensity
H
LysoTracker
Nucleus
I
LysoTracker per cell
****

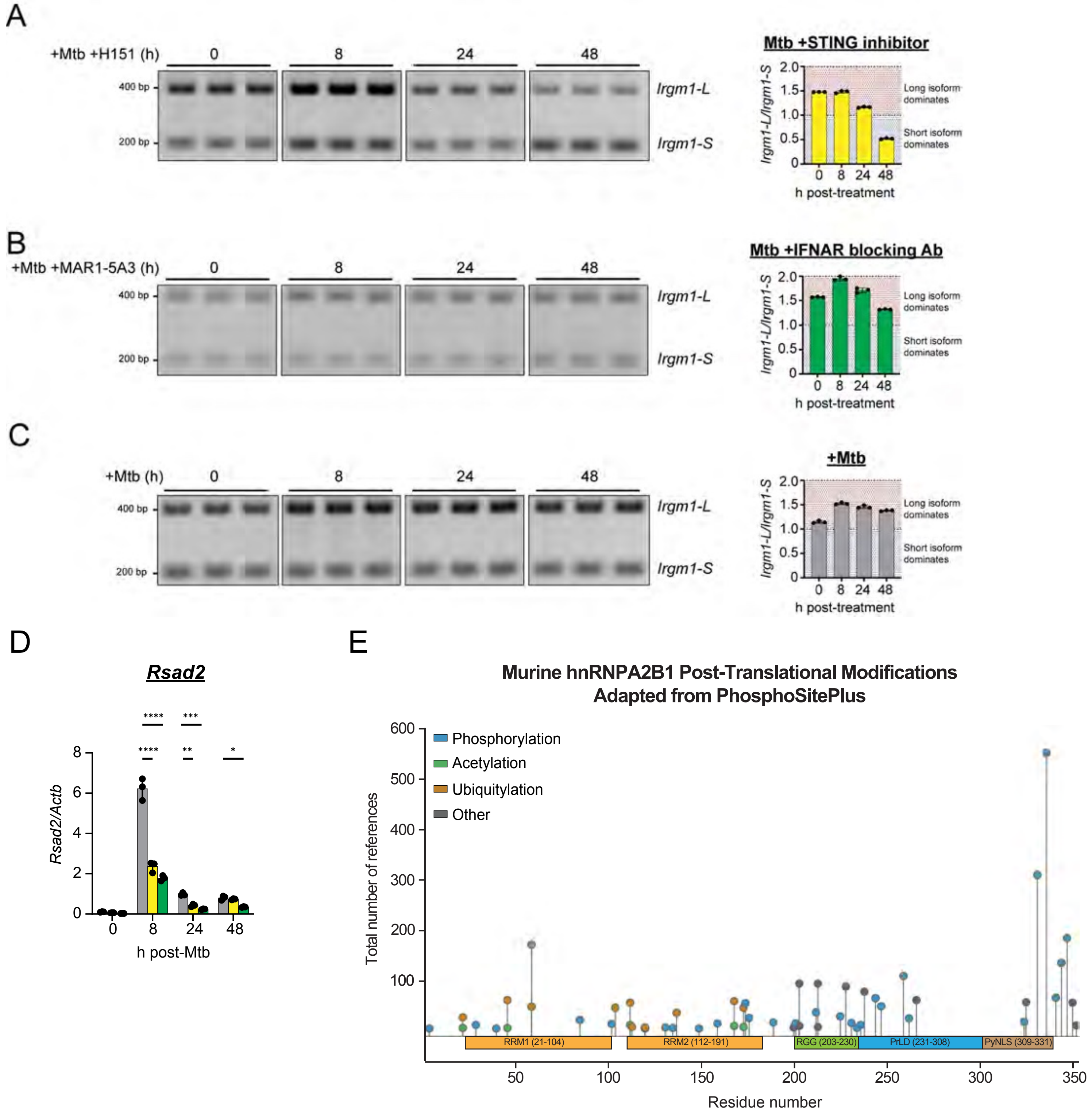
A
+Mtb +H151 (h)
0
8
24
48
400 bp
200 bp
Irgm1-L
Irgm1-S
Mtb +STING inhibitor
Irgm1-L/Irgm1-S
2.0
1.5
1.0
0.5
0
Long isoform dominates
Short isoform dominates
0 8 24 48
h post-treatment
B
+Mtb +MAR1-5A3 (h)
0
8
24
48
400 bp
200 bp
Irgm1-L
Irgm1-S
Mtb +IFNAR blocking Ab
Irgm1-L/Irgm1-S
Long isoform dominates
Short isoform dominates
0 8 24 48
h post-treatment
C
+Mtb (h)
0
8
24
48
400 bp
200 bp
Irgm1-L
Irgm1-S
+Mtb
Irgm1-L/Irgm1-S
Long isoform dominates
Short isoform dominates
0 8 24 48
h post-treatment
D
Rsad2
****
***
****
**
*
Rsad2/Actb
8
6
4
2
0
0 8 24 48
h post-Mtb
E
Murine hnRNPA2B1 Post-Translational Modifications
Adapted from PhosphoSitePlus
Phosphorylation
Acetylation
Ubiquitylation
Other
Total number of references
600
500
400
300
200
100
RRM1 (21-104)
RRM2 (112-191)
RGG (203-230)
PrLD (231-308)
PyNLS (309-331)
50
100
150
200
250
300
350
Residue number

Table S1

Differential gene expression in WT BMDMs (uninfected) vs. 8h post-Mtb infection.

Table S2

Local splicing variations (LSVs) identified by MAJIQ in WT BMDMs (uninfected) vs. 8h post-Mtb infection.

Table S3

Differential gene expression in WT BMDMs 8h post-Mtb infection vs. A2B1 KO BMDMs 8h post-Mtb infection.

Video S1

Live-cell fluorescence microscopy of E. coli (mCherry, red), LysoTracker (green), and nucleus (blue) 0 to 68 minutes post-E. coli infection in GFP, Irgm1-long, and Irgm1-short iBMDMs.
